# Microbial Community Dynamics During Lake Ice Freezing

**DOI:** 10.1038/s41598-019-42609-9

**Published:** 2019-04-17

**Authors:** Timothy M. Butler, Anna-Catharina Wilhelm, Amber C. Dwyer, Paige N. Webb, Andrew L. Baldwin, Stephen M. Techtmann

**Affiliations:** Department of Biological Sciences, Michigan Technology University, Houghton, MI USA

**Keywords:** Water microbiology, Microbial ecology

## Abstract

Many freshwater environments experience dramatic seasonal changes with some systems remaining ice-covered for most of the winter. Freshwater systems are also highly sensitive to environmental change. However, little is known about changes in microbial abundance and community composition during lake ice formation and times of persistent ice cover. The goal of this study is to characterize temporal dynamics of microbial communities during ice formation and persistent ice cover. Samples were collected in triplicate, five days per week from surface water in the Keweenaw Waterway between November and April. Environmental conditions along with microbial abundance and microbial community composition was determined. Distinct community composition was found between ice-free and ice-covered time periods with significantly different community composition between months. The microbial community underwent dramatic shifts in microbial abundance and diversity during the transitions into and out of ice cover. The richness of the microbial community increased during times of ice cover. Relatives of microbes involved in nitrogen cycling bloomed during times of ice cover as sequences related to known nitrifying taxa were significantly enriched during ice cover. These results help to elucidate how microbial abundance and diversity change over drastic seasonal transitions and how ice cover may affect microbial abundance and diversity.

## Introduction

Microbes in aquatic environments are drivers of many biogeochemical cycles. Many temperate freshwater ecosystems experience dramatic seasonal variation with some environments remaining ice-covered for most of the winter^1^. These strong perturbations have the potential to dramatically alter the microbial community composition during times of ice cover. Recent advances in sequencing technologies have provided unprecedented insights into microbial community diversity and dynamics. However, much still remains to be known about the dynamics of microbial communities during times of ice formation and persistence of lake ice.

While lakes and other freshwater systems represent a small portion of the total water on earth, they are integral to ecosystem function and biogeochemical cycling. Microbial community composition, activity, and function have been shown to change seasonally in freshwater settings^2^. Despite the dramatic change in environmental conditions from ice-free to ice-covered times, there is limited information regarding the impact of ice cover on microbial community composition in freshwater environments. Changes in microbial activity during ice-covered periods have implications on the ecosystem function and biogeochemical processes in these settings^2–4^.

Factors such as temperature, salinity, and dissolved oxygen may all act as limiting constraints on microbial activity and thus impact microbial diversity. During times of ice cover there are decreased temperatures, limited nutrient inputs, and low light levels^1^. Temperature is known to affect rates of biological reactions^5^. As temperatures are lower during times of ice cover compared to ice-free periods, it is expected that microbial activity would be lower. However, many microbes including psychrophilic and psychrotolerant microbes have adapted for growth under cold conditions and maintain high enzymatic activities and growth rates^6^.

As nutrients are often the limiting factor for microbial growth in these systems, the altered nutrient regime that occurs during times of ice cover may select for distinct populations capable of growth under nutrient limited conditions^1,7^. Additionally, as ice and snow accumulate at the surface of the lake, the extent of penetration of photosynthetically active radiation (PAR) into the underlying water is decreased^8^. This decrease in PAR can impact the activity of phototrophs during times of ice cover, and alter the primary productivity during ice cover. Despite lower light levels during ice cover, many systems experience routine under ice algal blooms, which have been shown to dramatically alter the microbial community composition following the algal bloom^9,10^. Additional work has shown that the extent of ice cover can strongly affect the phytoplankton and bacterial communities^11^.

Microbial communities are also known to be highly dynamic, with community changes on the timescales of days to months to seasons. Seasonal changes in microbial community composition have been studied in the oceans^12–15^, lakes^2,16^, rivers and streams^17,18^. These reports indicate that there was a distinct microbial community composition in different seasons. In many cases, this seasonal variation exhibits reproducible and predictable patterns in both the oceans and lakes^12,13,15,17–19^. Higher resolution variability on a day-to-day basis has been more finely investigated in marine settings^15,20^ with a few time series studies in freshwater systems^21^. These studies in the oceans have shown that there were dramatic and rapid shifts in the marine microbial community composition following seasonal change and in the days following phytoplankton blooms^15,20^. Time series of microbial community composition in freshwater lakes has shown that there is surprising variation in the microbial community between years^21^. These studies have also demonstrated that extrinsic factors such as environmental conditions play a role in determining the microbial community composition and providing some synchrony to the system^22^. In many freshwater and coastal systems, seasonal differences have been linked to differences in organic matter and nutrient inputs^17^. Biological interactions were also shown to be important in controlling the bacterial community composition of several temperate lakes during a time series study^22^. Despite our growing knowledge of temporal variation in freshwater microbial communities, very little is known about the stability of freshwater microbial communities on daily timescales, especially during the formation and persistence of lake ice.

The goal of this study was to understand the temporal dynamics of microbial community diversity before, during, and after ice-cover using high-resolution time series sample collection in a freshwater lake. The fresh water system chosen was the Keweenaw Waterway located in the Upper Peninsula of Michigan adjacent to Michigan Technological University. The Keweenaw Waterway is primarily fed by Portage Lake with some inputs from Lake Superior^23^. The Keweenaw Waterway experiences ice cover for approximately 3 months out of the year. Water samples were collected on a daily basis enabling a detailed look at the extent of change that a microbial community undergoes during this dramatic seasonal change. We expected there would be limited change in the microbial community during times of ice cover due to the stability of environmental conditions. We additionally, expected that microbial abundance and diversity would decrease during winter months. This study advances our understanding of the microbial community dynamics and diversity in freshwater systems that experience seasonal ice cover and opens the way for more targeted studies investigating the biogeochemical implications of these changes under lake ice.

## Results

### Environmental conditions stabilize during ice cover

**Figure 1:**
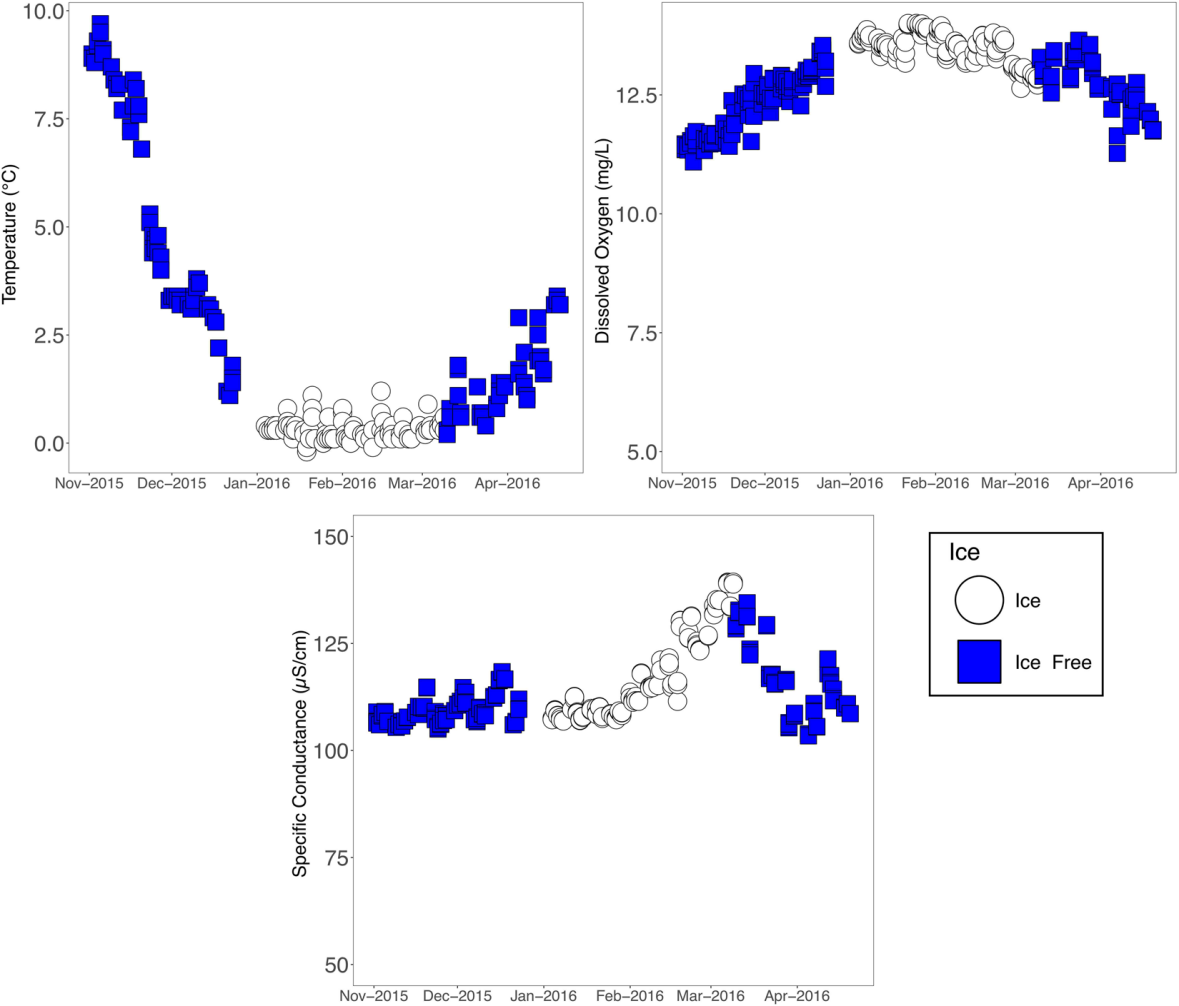
Environmental conditions. (**A**) Temperature changes observed throughout the time series. Ice-free samples are shown in blue and ice-covered samples are shown in white. (**B**) Dissolved oxygen measurements across the time series. (**C**) Specific conductivity across the time series.

Water temperature along with other physical and chemical measurements (temperature, conductivity, total dissolved solids (TDS), dissolved oxygen, pH, and ORP) were measured from surface water at the time of sampling during this time series (Supplemental Materials). The temperature consistently dropped until ice formation, at which point, the surface water temperature remained constant throughout the winter at approximately 0 °C (Fig. 1A). A slight increase in surface water temperature was observed in mid-December 2015. Dissolved oxygen in the surface waters also remained high throughout the study period (Fig. 1B). Conductivity remained fairly constant with a peak occurring at the time of ice melt and a rapid decline after ice melt (Fig. 1C). Other conditions including pH and ORP remained fairly constant throughout the study (Supplemental Materials).

### Changes in microbial abundance across the transition from fall to winter to spring

**Figure 2:**
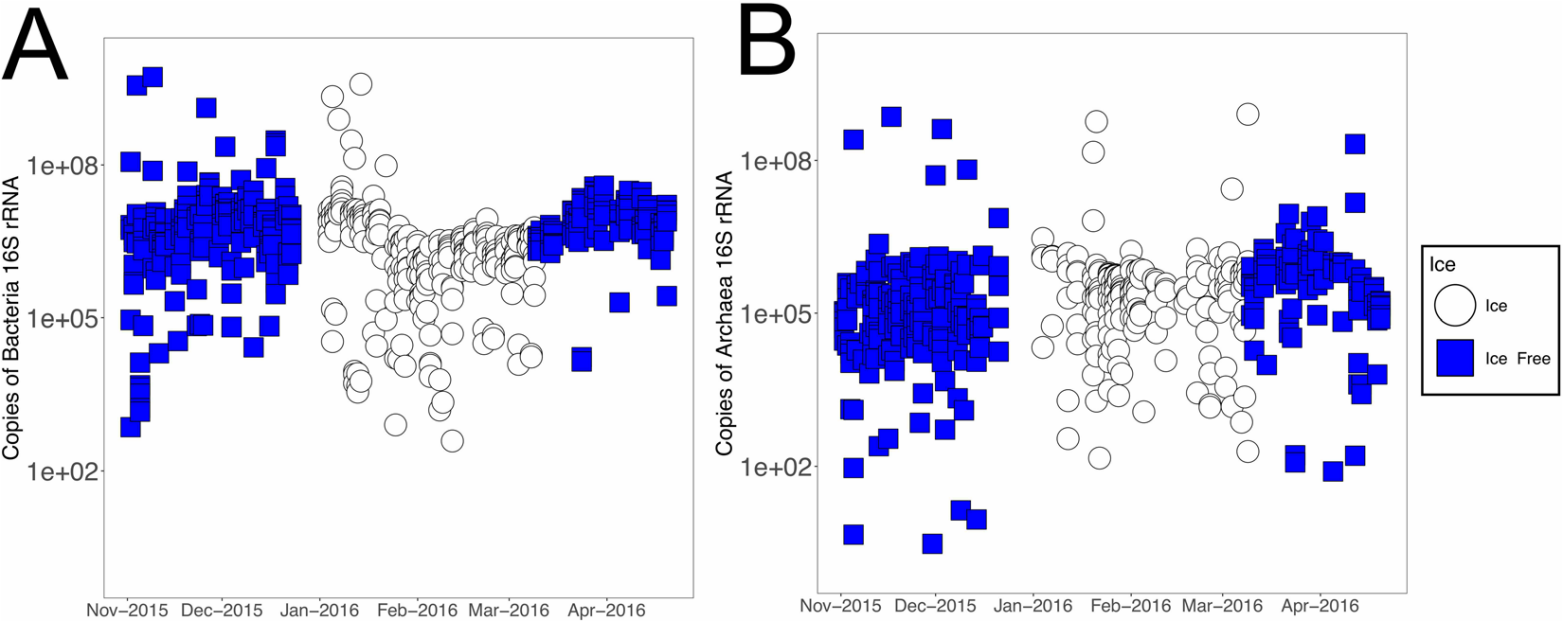
Bacterial and archaeal 16S rRNA gene abundance. (**A**) Abundance of bacterial 16S rRNA genes across the time series. (**B**) Abundance of archaeal 16S rRNA genes across the time series. Ice-free sample are shown in blue and ice-covered samples are shown in white.

To determine the impact of ice cover on microbial abundance, qPCR was used to measure abundances of archaea and bacteria across this time-series. Primers targeting the 16S rRNA gene were used to quantify the abundance of bacteria and archaea (Fig. 2 and Supplemental Materials). The average copies of archaeal 16S rRNA genes across the time series were found to be 7.24 × 10^6^ copies per ml of lake water, and the average abundance of bacterial 16S rRNA gene copies across the time series were found to be 8.52 × 10^7^ copies per ml. The average abundance of archaea during ice-free times was 6.35 × 10^6^ copies ml^−1^ whereas in ice-covered samples it was 8.62 × 10^6^ copies ml^−1^. The average bacterial abundance in ice-free conditions was 1.26 × 10^8^ copies ml^−1^ and during ice cover was 3.3 × 10^7^ copies ml^−1^. A Kruskal-Wallis test was performed on log transformed abundance data to determine if there was a significant difference in the bacterial and archaeal abundance in ice-free compared to ice-covered samples (Table 1). The average bacterial abundance varied significantly between ice-free and ice-covered conditions (Kruskal Wallis test p-value < 2.2 × 10^−16^, chi-squared 50.86, degrees of freedom = 1) (Fig. 2). Abundance of archaea was not significantly different between ice-free and ice-covered time periods. Despite the significance of this difference, there was less than a log change in the copies of the bacterial 16S rRNA gene in ice-covered and ice-free samples. Bacteria and archaeal abundance followed different trends with bacterial abundance decreasing during times of ice cover whereas there was a slight increase in archaeal abundance during times of ice cover. In particular, bacterial abundance decreased most substantially in the month of February and responded with an increase of almost one log by April (Supplementary Figs 1 and 2). In contrast the lowest archaeal abundance was observed in November and peaked in March.

**Table 1:** Statistics comparing prokaryotic abundance between ice-free and ice-covered conditions using Kruskal-Wallis test.

Comparison	p-value	Chi-squared	Degrees of Freedom
Archaea Ice-Free versus Ice	0.07755	3.1156	1
Bacteria Ice-Free versus Ice	<2.2 × 10^−16^	50.86	1

### Increase in phylogenetic diversity during ice cover

**Figure 3:**
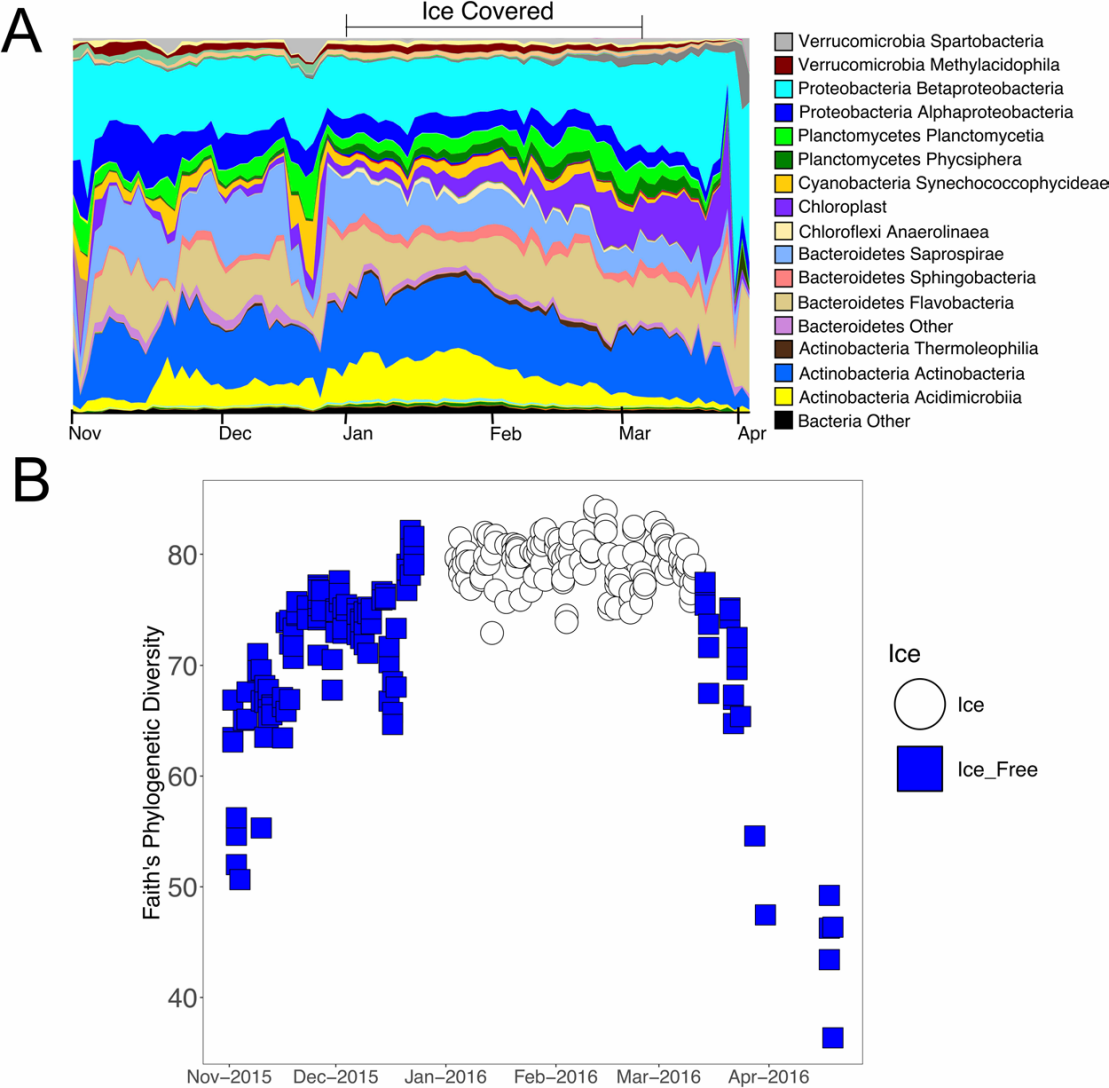
Microbial diversity across the timeseries. (**A**) Taxonomic area chart of microbial orders. Relative abundance of microbial orders are shown as different colors across the timeseries. Orders with abundance of less than 0.1% were grouped together in the other category. The corresponding colors for each microbial order are shown in the legend. (**B**) Faith’s Phylogenetic Diversity as a function of time. Samples collected during ice-free times are shown in blue squares. Samples collected during ice-covered times are shown in white circles.

The 16S rRNA gene was sequenced for each sample collected during this time series. The relative abundance of various phyla varied across the time series, with a distinct community present during times of ice cover (Fig. 3A) Faith’s Phylogenetic diversity was used to assess the phylogenetic diversity within each sample. Phylogenetic diversity increased during the fall with a maximum occurring at the same time as ice formation (Fig. 3B). This high phylogenetic diversity persists throughout the time of ice cover and then decreases upon ice melt to levels seen during the pre-ice period. This increase in diversity during times of ice cover was statistically different from the phylogenetic diversity during ice-free periods (Kruskal-Wallis p-value < 0.00001, Chi-squared = 150.33, degrees of freedom = 1) (Table 2). Additionally, there was a significant increase in richness (Observed species and Shannon) during times of ice cover (Supplementary Figs 3, 4 and 5 and Supplementary Table 3).

**Table 2:** ANCOVA analysis of rates of change for time-decay analysis.

	Fall Ice-Free	Ice	Spring Ice-Free
Fall Ice-Free		**<2.2** × **10**^**−16**^	**<2.2** × **10**^**−16**^
Ice	382.55		**0.03157**
Spring Ice-Free	73.02	4.6249	

Upper triangle represents p-values and lower triangle is F value for ANCOVA analysis.

### A distinct microbial community is established during times of ice cover

**Figure 4:**
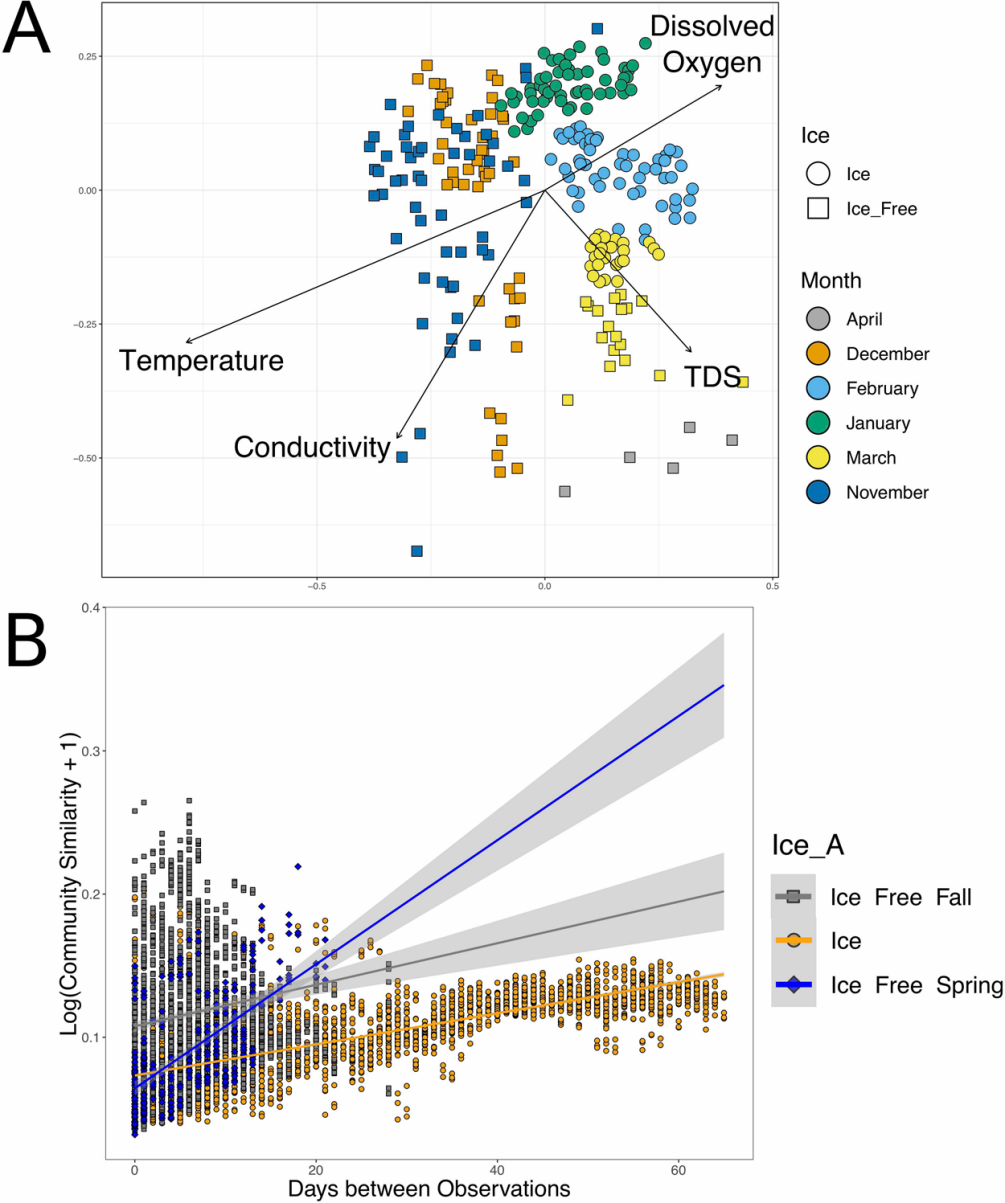
Microbial community composition changes during the time series. (**A**) Non-metric multidimensional scaling (NMDS) of weighted unifrac distances. The shape of the points corresponds to the ice condition and the color corresponds to month of collection. Stress for this plot is 0.1878. Environmental factors were fit to the NMDS and factors that significantly fit the data are shown as vectors. (**B**) Time-decay analysis of weighted-unifrac distances. Pairwise comparison of community distances versus number of days between treatment. Data was fit to a linear regression. Confidence regions are represented as gray shading. Ice-covered samples are shown in orange. Ice-free communities in the fall are shown in gray, and ice-free communities in the spring are shown in blue.

Multivariate analysis was performed in order to understand changes in the microbial community that occur during times of ice cover. Non-metric multidimensional scaling (NMDS) of weighted unifrac distances indicated that a distinct community of microbes existed in the water during times of ice cover relative to ice-free samples (Fig. 4A). PERMANOVA analysis on weighted unifrac distances indicated that there was a significant difference in the microbial community composition during times of ice-cover relative to ice-free conditions (PERMANOVA p-value = 0.001, F statistic = 44.927, R^2^ = 0.1488, degrees of freedom = 1). Furthermore, there are temporal changes in the microbial community that occurred throughout the year with a significant difference in the microbial community composition between months of the year (PERMANOVA p-value = 0.001, F statistic = 41.923, R^2^ = 0.45311, degrees of freedom = 5). To better understand the role of environmental conditions in explaining the microbial diversity, we fit the measured environmental variables to the NMDS of weighted unifrac distances (Fig. 4A). Temperature, conductivity, dissolved oxygen, and Total Dissolved Solids (TDS) all fit the data significantly (p-value = < 0.01). Temperature and conductivity strongly impacted the fall ice-free samples whereas dissolved oxygen was highest in the ice-covered samples and TDS was in the direction of the spring ice-free samples. CCA analysis showed similar impact of these environmental factors on the microbial community composition (Supplementary Fig. 6). Analysis of the R^2^ values for various comparisons indicated that ice cover alone was able to explain about 7% of the variance in the microbial community composition (adjusted R^2^ = 0.0773). The environmental conditions (temperature, conductivity, TDS and dissolved oxygen) explained about 17% of the variance (adjusted R^2^ = 0.174).

The clustering of the samples on the NMDS plot indicated that during ice-free periods there was a high amount of variability on a day-to-day basis in community composition (Fig. 4A). This finding was also supported with time-decay analysis (Fig. 4B). Furthermore, the microbial community from samples collected during times of ice cover clustered more tightly together, which suggests that the microbial community is more stable during times of ice cover from day-to-day. Despite the apparent stability of the microbial community during ice-cover, there were significant distinctions in the microbial community composition from month to month during ice-covered periods. Time-decay analysis demonstrates that ice-covered communities show a very slow rate of change relative to the ice-free samples (Fig. 4B). The rates of change were shown to be significantly different between ice-covered and ice-free conditions (ANCOVA p-value < 2.2e-16, F value = 224.29, degrees of freedom = 2). During the ice free periods, there were a few of times where there were abrupt changes in the microbial community composition. These changes to some extent occur at times of prolonged high winds (Supplementary Fig. 8).

To determine which species were differentially abundant between ice-free and ice-covered samples, a statistical analysis known as MetagenomeSeq was applied^24,25^. This test is designed to normalize the sequencing data and determine taxa that are differentially abundant between different conditions. Eighteen orders were significantly enriched in samples collected from ice-free conditions, whereas thirty-eight orders were enriched in ice-covered conditions relative to ice-free conditions (Tables 3 and 4).

**Table 3:** Microbial orders enriched in ice-free conditions with greater than 2-fold enrichment.

Order Enriched in Ice-Free conditions	Average Abundance Ice-Free	Average Abundance Ice	Adjusted P values
Armatimonadetes [Fimbriimonadia] [Fimbriimonadales]	0.02%	0.00%	1.37E-11
Cyanobacteria Chloroplast Haptophyceae	2.55%	0.34%	0.000000534
Verrucomicrobia Verrucomicrobiae Verrucomicrobiales	0.77%	0.14%	4.61E-16
Planctomycetes vadinHA49 DH61	0.04%	0.01%	5.78E-08
Cyanobacteria Chloroplast Cryptophyta	1.14%	0.33%	0.0000186
Proteobacteria Alphaproteobacteria Rhodobacterales	0.25%	0.08%	0.000000149
Proteobacteria Alphaproteobacteria Sphingomonadales	1.90%	0.63%	5.21E-13
Proteobacteria Alphaproteobacteria Caulobacterales	1.22%	0.41%	2.2E-09
Cyanobacteria Chloroplast Stramenopiles	1.91%	0.88%	0.000454789
Armatimonadetes Armatimonadia Armatimonadales	0.12%	0.06%	1.2E-17
Cyanobacteria Chloroplast	0.02%	0.01%	0.007421175

**Table 4:** Microbial orders enriched in ice-covered conditions with greater than 2-fold enrichment.

Order enriched in ice-covered conditions	Average Abundance Ice-Free	Average Abundance Ice	Adjusted P values
Planctomycetes Phycisphaerae Phycisphaerales	1.23%	2.66%	7.68E-30
Chloroflexi Anaerolineae H39	0.06%	0.86%	9.9E-28
Cyanobacteria Synechococcophycideae Pseudanabaenales	0.02%	0.19%	1.38E-27
Actinobacteria Thermoleophilia Gaiellales	0.09%	0.48%	1.03E-25
Nitrospirae Nitrospira Nitrospirales	0.05%	0.32%	2.17E-22
Actinobacteria Thermoleophilia Solirubrobacterales	0.11%	0.47%	3.24E-20
Verrucomicrobia Opitutae Opitutales	0.20%	0.48%	6.09E-18
Cyanobacteria; Other; Other	0.04%	0.13%	7.95E-17
Proteobacteria Betaproteobacteria Nitrosomonadales	0.07%	0.20%	3.86E-16
Acidobacteria Holophagae Holophagales	0.29%	0.60%	8.61E-16
Cyanobacteria Chloroplast Chlorophyta	1.82%	4.57%	1.58E-15
Thaumarchaeota Cenarchaeales	0.01%	0.02%	4.78E-14
Actinobacteria; Other; Other	0.14%	0.45%	3.32E-13
Planctomycetes; Other; Other	0.00%	0.02%	1.72E-11
Gemmatimonadetes Gemmatimonadetes KD8-87	0.00%	0.03%	3.66E-11
Proteobacteria Alphaproteobacteria; Other	0.08%	0.16%	7.2E-11
Proteobacteria Gammaproteobacteria Chromatiales	0.01%	0.02%	7.29E-11
Actinobacteria Acidimicrobiia Acidimicrobiales	3.33%	7.93%	0.000000372
Cyanobacteria Synechococcophycideae; Other	0.01%	0.04%	0.0000168
TM7 SC3	0.07%	0.15%	0.0000417
Chloroflexi Ktedonobacteria TK10	0.02%	0.07%	0.000285764

**Figure 5:**
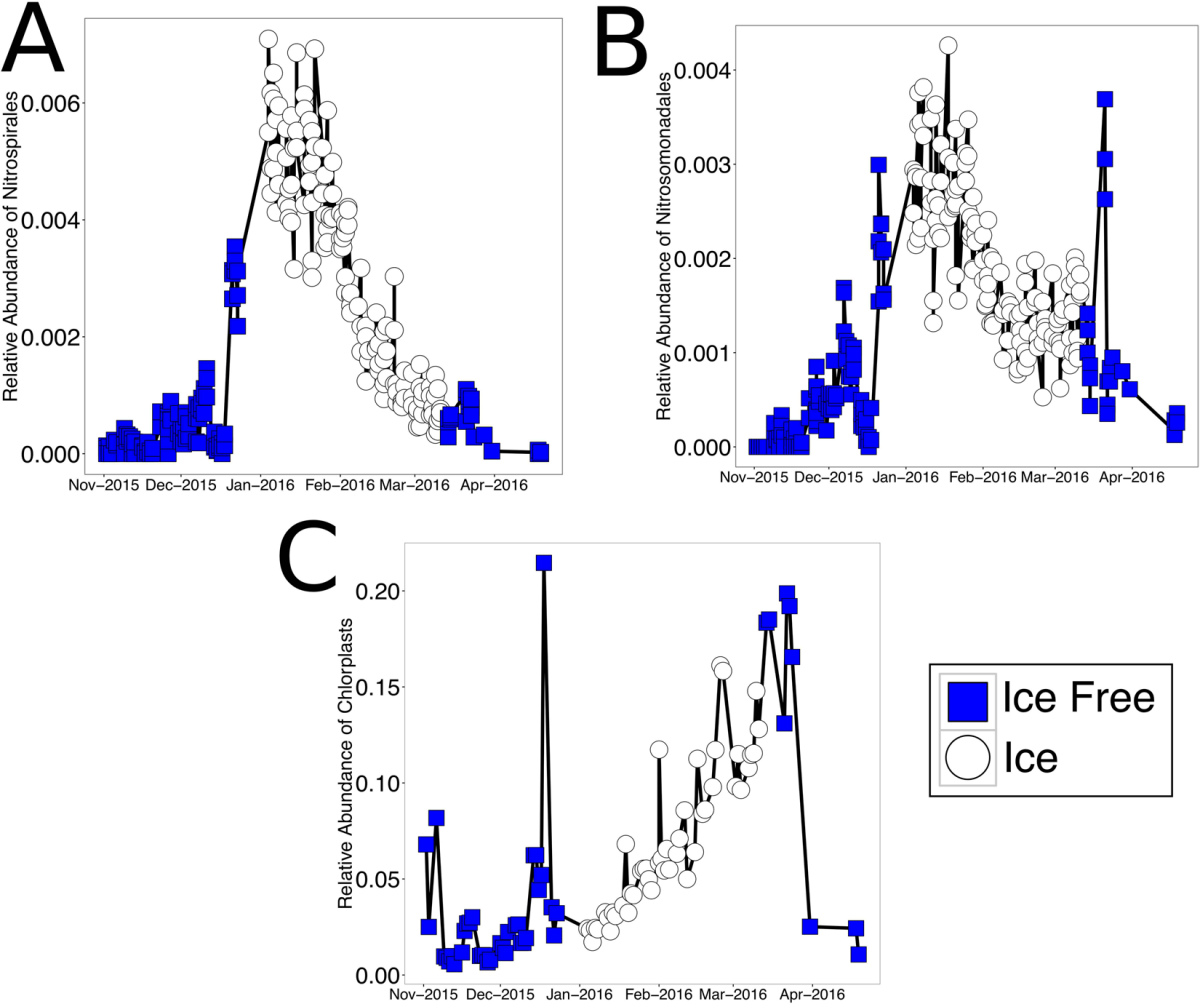
Differentially abundant taxa. Select taxa that are significantly different between ice-free and ice-covered states. (**A**) Relative abundance of sequences classified as Nitrospira as a function of time. (**B**) Relative abundance of sequences classified as Nitrosomonadales as a function of time. (**C**) Relative abundance of sequences classified as Chloroplasts as a function of time.

Members of the Burkholderiales, Verrucomicrobales, and Caulobacteriales, were some of the most significantly enriched orders under ice-free conditions. Verrucomicrobiales were enriched 5 fold under ice-free conditions, while Caulobacteriales were enriched 3 fold, and Burkhoderiales were enriched 1.9 fold in ice-free conditions. Both ammonia oxidizing bacteria (AOB – Nitrosomonadales) and ammonia oxidizing archaea (AOA – Thaumarchaeota) were enriched during ice cover. Additionally, relatives of nitrite oxidizing bacteria of the order Nitrospirilales were enriched more than 6-fold during times of ice cover (Fig. 5).

### Under-ice algal bloom

A substantial increase in reads classified as chloroplast sequences was observed during times of ice cover leading up to ice melt (Fig. 5). Reads assigned to chloroplasts were typically less than 5% of the recovered reads in November and December. However, there was a steady increase in chloroplast sequences during times of ice cover. The percent of chloroplast sequences during this under ice bloom peaked at (18%) just after ice melt in March and subsequently dropped off to pre-ice levels in April. For the most part, these sequences could not be further classified to detail from which photosynthetic eukaryotes these chloroplasts were derived. This finding would suggest that there was an under-ice algal bloom that occurred in this water body during the ice-covered season.

## Discussion

Here we sought to investigate the dynamics of the microbial community of surface water across dramatic seasonal changes in the Keweenaw Waterway. The Keweenaw Waterway is a highly dynamic system where movement of water can be influenced by wind driven currents^23^. The environmental conditions of the surface water exhibited dramatic changes leading up to ice cover, but stabilized during times of ice cover. This stability is similar to the trends in temperature that were observed in Churchill *et al*.^23^. Temperature can affect microbial activity and abundances. There was a slight but statistically significant decrease in the bacterial abundance during times of ice cover (p-value < 2.2 × 10^−16^). Ice cover was present from early January to mid-March during the time of sampling in this study. During ice cover the temperature of the surface water stabilized at approximately 0 °C and the dissolved oxygen of the surface water stabilized at approximately 12.5 mg/L. This indicated that despite the limited gas exchange during times of ice cover, the surface water remained oxygenated, allowing for dominance of aerobic physiologies during the ice-covered time period.

Our results suggest that microbial biomass in the surface water showed some limited change between ice-free and ice-covered conditions. Previous studies have shown that bacteria cell numbers remain fairly constant throughout the year with blooms associated with thermal mixing^26^. Others have reported bacterial biomass is typically lower during ice cover^1^. The decrease in bacterial abundance observed in this study was slight (less than one log), suggesting that microbes in this lake are well adapted for cold conditions. This could be due to the consistent long periods of ice cover and the limited warming that occurs in this lake during the summer.

Despite the conditions found under ice, previous studies would suggest that these microbes are actively growing^1^. This active growth suggests the possibility for rapid change to brief or more gradual changes in environmental conditions or inputs of nutrients. This can be observed in the gradual changes that occur throughout the ice-covered times. Our results would suggest that the microbial community present during times of ice cover was more stable on a day-to-day basis compared to the ice-free periods. However, the microbial community present under ice exhibited gradual changes, which is in line with the previous studies that demonstrate an actively growing microbial community during times of ice cover and changes in the bacterial community composition during ice cover^26^. Part of this actively growing community appears to be algae. We observed a substantial increase in chloroplast sequences during the middle of ice cover and increasing until ice melt. This under ice algal bloom matches with other previously described blooms of under ice algae^9^. This bloom was quite slow in forming as the build-up lasted for almost 2 months – starting in mid-January not long after ice formation and continued to increase until ice melt in mid-March.

Interestingly, we observed an increase in the phylogenetic diversity of the microbes during the ice-covered conditions. Our initial hypothesis, that the diversity would decrease, was based on the previous observations that microbial biomass decreases during times of ice cover coupled with the fact that the conditions found under ice cover are much more selective for growth (low temperature, low light, limited inputs of nutrients). Therefore, it was surprising to observe an increase in the both richness and evenness during times of ice cover. This increase in diversity could be explained through a couple different ways. The increased diversity could be due to limited grazing that occurs during times of ice cover^27^. The decreased grazing would relieve some of the top down controls on the microbial community composition and allow for a more diverse community to proliferate without the control of grazing that occurs during warmer months. The increase in phylogenetic diversity could also be explained by alternation in the nutrient inputs into the system. It is possible that during warmer months the increased temperatures or nutrients allow for particular taxa to dominate the system. The seasonal changes that occurs during ice cover could select against these warm-adapted microbes and may allow for more access to the available nutrients, thus enabling the proliferation of a more diverse community. Alternatively, this increased diversity could be explained by the persistence of a large number of dormant microbes in this system. Our work was done on the level of DNA, which often does not represent the actively metabolizing microbes in the system^28^. Additionally, there are studies that have suggested that microbial dormancy can be a mechanism by which microbial diversity is maintained in low nutrient environments^29^. In Jones and Lennon (2010), a dormant taxon is considered to be one which is detectable on the DNA level, but is not detectable at the RNA level. It is therefore possible that the increased diversity reflects an expanding number of detectable, but dormant taxa that are now observable due the decrease in the dominant taxa found in warmer months. Previous work has demonstrated that the percent of dormant organisms in a lake was in part affected by the nutrient loads^29^. This previous work demonstrated that, in some nutrient-poor systems, up to 40% of the taxon richness is derived from dormant cells. However, we did not measure the active community in these samples and thus cannot confirm that this observed increase in diversity is indeed due to dormancy. Furthermore, the dynamic nature of the Keweenaw Waterway leads to mixing of water from Lake Superior and Portage Lake. While it is possible that this mixing could contribute to some of the increased diversity, the stability of this elevated diversity over many months suggests that this is not a local or ephemeral phenomenon impacted by transient changes in mixing and water movement. Additionally, Churchill, *et al*. (2004) observed little mixing during times of ice cover, which supports the concept that the microbial community compositional changes are stable and not impacted by wind driven mixing^23^. Therefore, it is possible that this increased diversity could be due to a number of factors including decreased grazing, competition, or dormancy, which should be further investigated in future studies.

To parse out the differences observed on the overall community, differentially abundant operational taxonomic units (OTUs) were identified. A number of taxa were shown to be differentially abundant between ice-free and ice-covered periods. Many of taxa that showed the highest fold change during times of ice cover were related to microbes known to be involved in then nitrogen cycle. In particular, sequences related to microbes involved in the process of nitrification were enriched during times of ice cover. Members of the Nitrospirales, Nitrosomonadales, and Thaumarchaeota were all significantly enriched in the ice-covered conditions. A recent study suggested that ice duration drives accumulation of nitrate in north temperate lakes^30^. This increased nitrate was proposed to be associated with nitrification, which could decreased oxygen levels in deeper waters under ice^31^. In our study, we observed an increase in sequences related to nitrifying bacteria in the surface water below the ice. However, we did not measure nitrification as part of this study. While other studies have shown increased nitrification under ice, more work is needed to confirm the role of these organisms related to nitrifiers in nitrogen cycling under ice in the Keweenaw Waterway. Our work only examined the microbial community in the surface water using 16S rRNA gene sequencing. We did not observe decreased oxygen levels, which may be more prevalent in deeper waters. It would be important to follow up this work to link these putative nitrifiers with increase rates of nitrification under ice observed in other under ice lake systems.

## Conclusion

This study aimed to understand how microbial abundance and diversity changes over drastic seasonal transitions, and how ice cover affects microbial abundance and diversity. Molecular techniques and next generation sequencing provided a detailed survey of the microbial community in the Keweenaw waterway through a period of ice cover. Our results suggest that ice formation may cause shifts in the microbial community composition. The increase in diversity during times of ice cover suggests that the conditions during ice formation may be a strong constraint on the growth of some species and in return remove the constraint for growth of other species. Additionally, an increase in sequences related to taxa involved in nitrification during times of ice-cover suggests that ice cover may impact biogeochemical cycling and potentially have impacts on the nitrogen cycle. A better understanding of the impact of ice cover on biogeochemical cycling will be important for characterizing the impact of changes in the extent and duration of lake ice cover on biogeochemical fluxes. In the long-term there is need to characterize the temporal changes in microbial community composition and biogeochemical cycling in order to better understand how seasonal variations and ice cover impact ecosystem function.

## Materials and Methods

### Water Sampling

Triplicate water samples were collected five days per week from the surface of the Keweenaw Waterway. During times of ice cover, these samples were collected from directly below the ice. During sampling, *in situ* environmental variables were measured with a YSI proDSS sonde which measured temperature, dissolved oxygen (DO), conductivity, Total Dissolved Solids (TDS), ORP, and pH (Supplemental Table 1). Samples were collected from 2 November 2015 to 20 April 2016. Water samples were immediately transported to the lab (<10 minutes). Samples for microbial community analysis were collected by filtering 600 ml of water through a 0.2 µm PES filter, using a vacuum pump system. Filters were immediately stored at −80 °C.

### DNA Extraction

DNA was extracted from half of each filter using a modified Miller protocol^32^. Half of each filter was placed into a Lysing Matrix E tube (MP Biomedicals) along with 300 µl of Miller Phosphate buffer, 300 µl of Miller SDS lysis buffer, and 600 µl of Phenol:Chloroform:Isoamly alcohol (25:24:1). A process blank was also setup and subjected to all following steps of extraction without a filter. The tubes were homogenized in a FastPrep-24 bead-beater (MP Biomedicals) for 45 seconds at a speed of 5.5 m/s. To remove cell debris and filter material, the tubes were centrifuged at 10,000 × g for 5 minutes. 600 µl of aqueous supernatant was transferred to a new 2 ml tube along extracted with one volume of chloroform. Tubes were centrifuged at 10,000 × g for 5 minutes, then the aqueous phase was kept. Purification and concentration of recovered DNA was performed by adding two volumes of MoBio solution C4 to the aqueous phase. This was passed over a MoBio spin filter. The spin filters were then washed with 400 µl of MoBio C5 solution. Residual C5 was removed by centrifuging the empty tubes at 10000 × g for two minutes. DNA was eluted by two 30 µl additions of MoBio C6 solution. Final eluted environmental DNA was stored in −80 °C freezer. DNA concentrations were determined using NanoDrop spectrophotometer.

### qPCR

The abundance of bacterial and archaeal 16S rRNA was determined using qPCR. qPCR was performed on an StepOne Plus instrument (Applied Biosystems, Foster City CA). Six-point standard curves were performed in triplicate with concentrations ranging from 2 × 10^−4^ pM to 20 pM. The equation for the standard curve to convert C_T_ to pM of 16S rRNA genes was y = 16736(e^(−0.515x)^). The R^2^ of our standard curves was 0.97. Environmental DNA was diluted 1:10 to limit the impact of inhibitors from environmental DNA. To each reaction 1 µl of the diluted environmental DNA was used in 20 µl qPCR reactions. The copy numbers of bacterial 16S rRNA, archaeal 16S rRNA in environmental DNA were determined in duplicate for each sample. For bacterial 16S rRNA gene quantification, Bact341 and Uni519R primers were used according to the protocol described in Jorgenson *et al*.^33^. Archaeal 16S rRNA abundance was determined using Uni519R and Arch908R as described in Jorgenson *et al*.^33^. Standards and PCR reaction set up was similar to that described in Techtmann *et al*.^34^. Normality of the qPCR data was tested with the Shapiro-Wilk test for normality as implemented in R^35^. Since the data was not normally distributed, the Kruskal-Wallis one way analysis of variance was used to determine if there was a significant difference in the microbial abundances between ice-free and ice-covered periods.

### 16sS rRNA sequencing

The V4 and V5 hypervariable region of the 16S rRNA gene was amplified using Phusion DNA polymerase (Thermo Scientific, Waltham, MA) with universal primers 515F-Y and 926R^36^. These primers were able to amplify Bacterial and Archaeal 16S rRNA gene as well as many Eukaryotic 18S rRNA genes. The 16S rRNA gene was amplified by 25 cycles. The amplicon was purified using AxyPrep PCR Clean up magnetic beads (Axigen). Index sequences and sequencing adapters were added to each sample by an additional 8 cycle PCR as described in the Illumina 16S rRNA Metagenomic Library Preparation protocol. Sequencing was performed on the Illumina MiSeq according to the Illumina protocol for amplicon sequencing. The resulting DNA sequences were analyzed using the QIIME version 1.9.0-dev pipeline^37^. Paired-end raw reads were assembled using fastq-join^38^. The assembled sequences were demultiplexed and quality filtered in QIIME to remove reads with phred scores below 20 (-q 19). Chimera detection was then performed on assembled reads using VSEARCH^39^. The taxonomy for each read was assigned using RDP classifier^40^ retrained with SILVA release 123. Data was rarified to the lowest number of sequences in a sample (3052). Alpha diversity was calculated using the alpha_diversity.py command in QIIME. Faith’s phylogenetic diversity, Observed Species, and Shannon diversity were calculated from the rarified OTU table. Weighted and unweighted Unifrac distances as well as Bray Curtis dissimilarity were calculated from the rarified OTU table using the beta_diversity.py command in QIIME. Raw sequencing reads have been deposited in the SRA as accession number SRP159623.

### Statistical Analysis on 16S rRNA sequencing data

Normality of the alpha diversity data was tested with the Shapiro-Wilk test. The Shapiro-Wilk test for normality of the Faith’s Phylogenetic alpha diversity returned a p-value of less than 2.2 × 10^−16^, which indicates that the data is not normally distributed. Since the Faith’s Phylogenetic Diversity data was not normally distributed, we chose to use a Kruskal-Wallis test to test the hypothesis that there were significant differences in the alpha diversity between ice-free and ice-covered samples. To determine if there was a significant difference in the alpha diversity between months, a Kruskal-Wallis test was performed comparing differences in the Faith’s Phylogenetic diversity between months. A Dunn test was used as a post-hoc test to determine between which months there was a statistically significant difference. The Dunn test was performed using the dunn.test package in R^41^. P-values for the Dunn test were adjusted for multiple comparisons using the Bonferroni method.

To visualize differences in the community structure between ice states and between months, nonmetric multidimensional scaling (NMDS) was performed on the weighted unifrac distance matrix using the metaMDS command implemented in the vegan package^42^. The lowest stress configuration of 50 iterations was chosen. NMDS analysis with unweighted unifrac distances was also performed and is included in Supplementary Fig. 7. Environmental variables were fit to the NMDS plots using the envfit function in vegan. Factors that significantly fit the data were plotted as vectors. PERMANOVA analysis was used to identify if there were significant differences in community structure between ice states and months using the adonis function in the vegan package with 999 permutations^42^. To further understand the impact of environmental variables on explaining the data, a CCA was performed on the weighted unifrac distances. Co-linear environmental factors were removed. The role of Temperature, conductivity, TDS, and Dissolved oxygen was tested using the CCA. The CCA plot is shown in Supplementary Fig. 6. Redundancy analysis was used to understand that amount of variance explained by ice-cover and the other environmental variables. Rda was performed in R using the vegan package. The rarified OTU table was normalized using heilinger method. RDA was then performed against Ice cover or a table of scaled environmental variables. The R^2^ was then adjusted using the RsquareAdj function in vegan.

To determine rates of change in community composition a time-decay analysis was performed to identify how ice cover impacted rates of community change. This approach was used to further determine the rates of community composition change during times of ice cover compared to ice-free conditions. Weighted unifrac distances were determined for each sample in a pair-wise manner. The log of these pair-wise dissimilarities were then plotted based on the time between each comparison and regression line generated for each case. Rates of change can be determined through analyzing the slope of these regression lines. ANCOVA analysis was performed to determine if the differences in rates of change were significant between ice-free and ice-covered conditions.

A finer scale analysis on the level of OTUs was undertaken to examine how particular OTUs are affected by ice state. The MetagenomeSeq package^24,25^, as implemented in QIIME, was used to identify which OTUs that were differentially abundant between ice-states. This analysis was done on the non-rarified OTU table. OTUs that were significantly different (corrected p value < 0.05) in one state were considered to be differentially abundant.

## Supplementary information

Supplementary Materials

Dataset 1

## Data Availability

The sequencing datasets as part of this study are deposited at the Sequence Read Archive under accession number SRP159623.

## References

[1] Bertilsson S (2013). The under - ice microbiome of seasonally frozen lakes. Limnology and Oceanography.

[2] Wilhelm SW (2014). Seasonal changes in microbial community structure and activity imply winter production is linked to summer hypoxia in a large lake. FEMS microbiology ecology.

[3] Gammons CH (2014). Stable isotopes track biogeochemical processes under seasonal ice cover in a shallow, productive lake. Biogeochemistry.

[4] Ricão Canelhas, M., Denfeld, B. A., Weyhenmeyer, G. A., Bastviken, D. & Bertilsson, S. Methane oxidation at the water - ice interface of an ice - covered lake. *Limnology and Oceanography* **61** (2016).

[5] Hoppe HG (2008). Climate warming in winter affects the coupling between phytoplankton and bacteria during the spring bloom: a mesocosm study. Aquat Microb Ecol.

[6] De Maayer P, Anderson D, Cary C, Cowan DA (2014). Some like it cold: understanding the survival strategies of psychrophiles. Embo Rep.

[7] Kearns PJ (2016). Nutrient enrichment induces dormancy and decreases diversity of active bacteria in salt marsh sediments. Nature communications.

[8] Lundberg J (2007). Light tracking through ice and water—Scattering and absorption in heterogeneous media with PHOTONICS. Nuclear Instruments and Methods in Physics Research Section A: Accelerators, Spectrometers, Detectors and Associated Equipment.

[9] Bižić-Ionescu M, Amann R, Grossart H-P (2014). Massive regime shifts and high activity of heterotrophic bacteria in an ice-covered lake. PloS one.

[10] Dokulil MT, Herzig A (2009). An analysis of long-term winter data on phytoplankton and zooplankton in Neusiedler See, a shallow temperate lake, Austria. Aquatic Ecology.

[11] Beall BFN (2016). Ice cover extent drives phytoplankton and bacterial community structure in a large north-temperate lake: implications for a warming climate. Environmental Microbiology.

[12] Giovannoni SJ, Vergin KL (2012). Seasonality in ocean microbial communities. Science.

[13] Fuhrman JA, Cram JA, Needham DM (2015). Marine microbial community dynamics and their ecological interpretation. Nature Reviews Microbiology.

[14] Gilbert JA (2012). Defining seasonal marine microbial community dynamics. The ISME journal.

[15] Ward CS (2017). Annual community patterns are driven by seasonal switching between closely related marine bacteria. The ISME journal.

[16] Crump BC, Kling GW, Bahr M, Hobbie JE (2003). Bacterioplankton community shifts in an arctic lake correlate with seasonal changes in organic matter source. Applied and Environmental Microbiology.

[17] Crump BC, Hobbie JE (2005). Synchrony and seasonality in bacterioplankton communities of two temperate rivers. Limnology and Oceanography.

[18] Hullar MA, Kaplan LA, Stahl DA (2006). Recurring seasonal dynamics of microbial communities in stream habitats. Applied and Environmental Microbiology.

[19] Li J (2015). Annual periodicity in planktonic bacterial and archaeal community composition of eutrophic Lake Taihu. Scientific reports.

[20] Needham DM, Fuhrman JA (2016). Pronounced daily succession of phytoplankton, archaea and bacteria following a spring bloom. Nature microbiology.

[21] Linz AM (2017). Bacterial community composition and dynamics spanning five years in freshwater bog lakes. mSphere.

[22] Kent AD, Yannarell AC, Rusak JA, Triplett EW, McMahon KD (2007). Synchrony in aquatic microbial community dynamics. Isme J.

[23] Churchill JH, Kerfoot WC, Auer MT (2004). Exchange of water between the Keweenaw Waterway and Lake Superior: characteristics and forcing mechanisms. Journal of Great Lakes Research.

[24] Paulson, J. N., Pop, M. & Bravo, H. metagenomeSeq: Statistical analysis for sparse high-throughput sequencing. *Bioconductor package* **1** (2013).

[25] Paulson JN, Stine OC, Bravo HC, Pop M (2013). Differential abundance analysis for microbial marker-gene surveys. Nature methods.

[26] Pernthaler J (1998). Seasonal community and population dynamics of pelagic bacteria and archaea in a high mountain lake. Applied and Environmental Microbiology.

[27] Tulonen T, Kankaala P, Ojala A, Arvola L (1994). Factors Controlling Production of Phytoplankton and Bacteria under-Ice in a Humic, Boreal Lake. Journal of Plankton Research.

[28] Techtmann SM, Hazen TC (2016). Metagenomic applications in environmental monitoring and bioremediation. Journal of industrial microbiology & biotechnology.

[29] Jones SE, Lennon JT (2010). Dormancy contributes to the maintenance of microbial diversity. Proceedings of the National Academy of Sciences.

[30] Powers SM (2017). Ice duration drives winter nitrate accumulation in north temperate lakes. Limnology and Oceanography Letters.

[31] Powers S (2017). Nitrification contributes to winter oxygen depletion in seasonally frozen forested lakes. Biogeochemistry.

[32] Techtmann SM (2015). The unique chemistry of Eastern Mediterranean water masses selects for distinct microbial communities by depth. PLoS One.

[33] Jorgensen SL (2012). Correlating microbial community profiles with geochemical data in highly stratified sediments from the Arctic Mid-Ocean Ridge. P Natl Acad Sci USA.

[34] Techtman SM (2017). Comparison of Thaumarchaeotal populations from four deep sea basins. FEMS microbiology ecology.

[35] Team, R. C. R: A language and environment for statistical computing. (2013).

[36] Parada, A. E., Needham, D. M. & Fuhrman, J. A. Every base matters: assessing small subunit rRNA primers for marine microbiomes with mock communities, time series and global field samples. *Environmental microbiology* (2015). 10.1111/1462-2920.13023 26271760

[37] Caporaso JG (2010). QIIME allows analysis of high-throughput community sequencing data. Nature Methods.

[38] Ea-utils: “Command-line tools for processing biological sequencing data” (2011).

[39] Rognes T, Flouri T, Nichols B, Quince C, Mahé F (2016). VSEARCH: a versatile open source tool for metagenomics. PeerJ.

[40] Wang Q, Garrity GM, Tiedje JM, Cole JR (2007). Naive Bayesian classifier for rapid assignment of rRNA sequences into the new bacterial taxonomy. Applied and Environmental Microbiology.

[41] Dinno, A. dunn. test: Dunn’s test of multiple comparisons using rank sums. *R package version* **1** (2015).

[42] Oksanen, J. *et al*. Vegan: Community Ecology Package. *R package version 2.0*–*10*, http://cran.r-project.org/package=vegan (2013).

