# Supplementary material for: Microbial Community Dynamics During Lake Ice Freezing
Source: Sci Rep. 2019 Apr 17;9:6231. doi: 10.1038/s41598-019-42609-9 (PMC6470161; doi:10.1038/s41598-019-42609-9)
Supplement: Supplementary file 1 — Supplementary Materials [file 41598_2019_42609_MOESM1_ESM.pdf]

Microbial Community Dynamics During Lake Ice Freezing

Timothy M. Butler[1], Anna-Catharina Wilhelm[1], Amber C. Dwyer[1], Paige N. Webb[1], Andrew L. Baldwin[1], Stephen M. Techtmann[1]

[1] Department of Biological Sciences, Michigan Technology University, Houghton MI

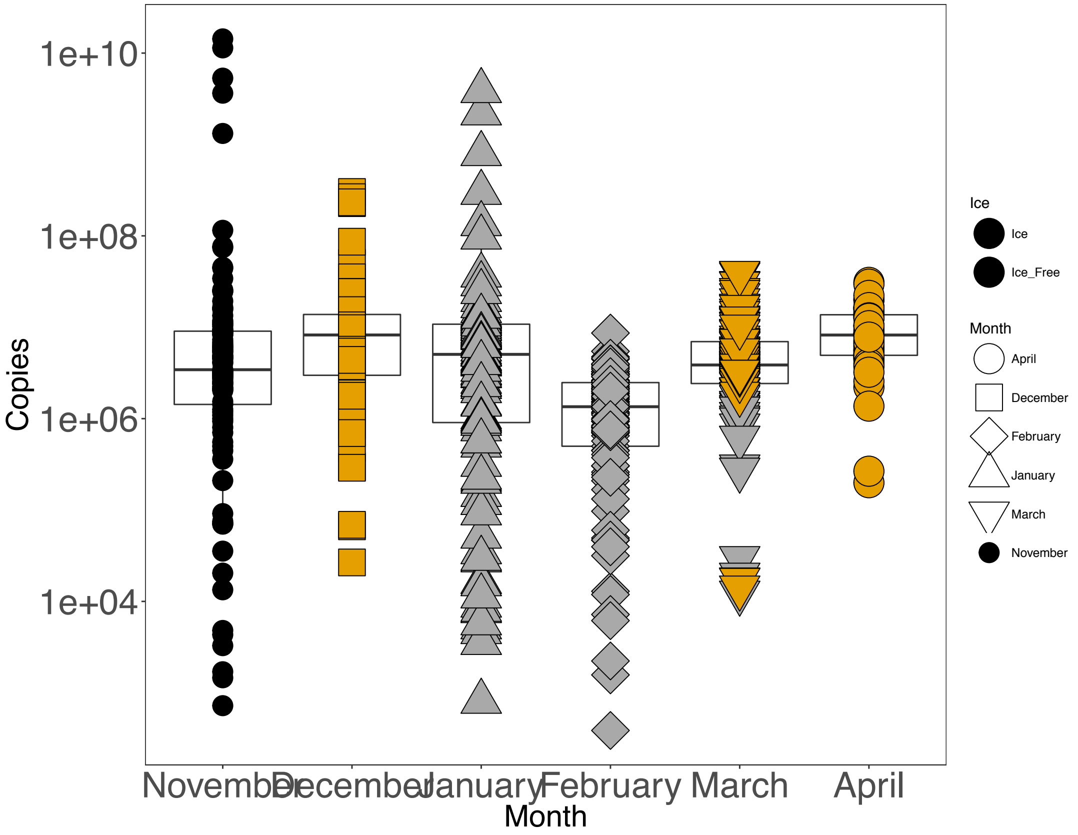

Supplemental Figure 1: Abundance of Bacterial 16S rRNA genes across the time series separated by month of sampling. Samples from ice-free conditions are shown in orange and samples from ice covered times are shown in gray

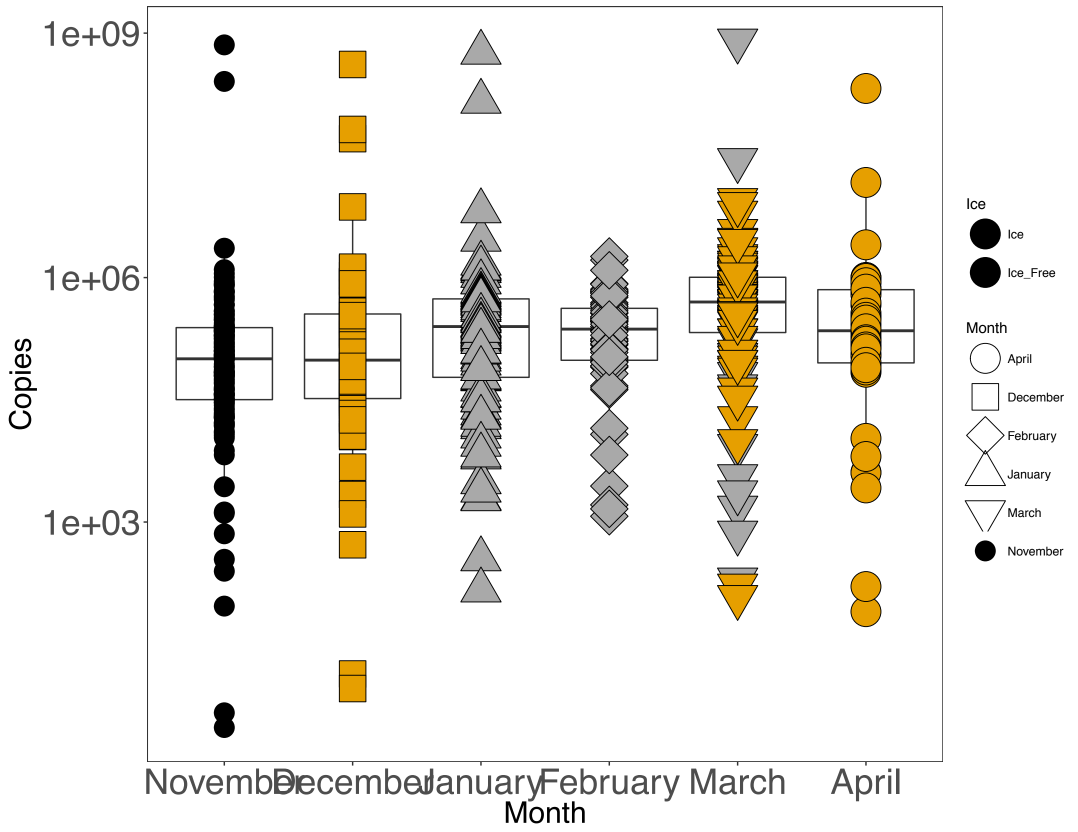

Supplemental Figure 2: Abundance of archaeal 16S rRNA genes across the time series separated by month of sampling. Samples from ice-free conditions are shown in orange and samples from ice covered times are shown in gray

Supplemental Table 1: Kruskal-Wallis test comparing the log bacterial copies by months returned the following statistics: p-value = < 2.2 x 10^{-16}, chi-squared = 115.88, degrees of freedom = 5. Dunn test comparing log of bacterial abundance for each month. P-values of adjusted for multiple comparisons using Bonferroni correction and shown in the upper triangle and significant comparisons are shown in bold. Z stats are shown in the lower triangle in italic.

Month - log Copies - Bact	November	December	January	February	March	April
November		**0.01213**	1	**<0.00001**	1	**0.00398**
December	*3.349*		**0.03101**	**<0.00001**	**0.0219**	1
January	*0.293*	*3.080*		**<0.00001**	1	**0.00945**
February	*-6.105*	*9.266*	*-6.426*		**<0.00001**	**<0.00001**
March	*0.149*	*3.181*	*0.140*	*-6.210*		**0.00682**
April	*3.646*	*0.798*	*3.418*	*8.647*	*3.505*	

Supplemental Table 2: Kruskal-Wallis test comparing months returned the following statistics: p-value = 3.68×10^{-9}, chi-squared = 47.925, degrees of freedom = 5. Dunn test comparing log of archaeal abundance for each month. P-values of adjusted for multiple comparisons using Bonferroni correction and shown in the upper triangle and significant comparisons are shown in bold. Z stats are shown in the lower triangle in italic.

Month - log Copies - Arch	November	December	January	February	March	April
November		1	**0.0117**	0.0736	**<0.00001**	0.0521
December	*1.109*		0.967	1	**<0.00001**	1
January	*3.358*	*-1.848*		1	**0.0358**	1
February	*2.812*	*-1.537*	*-0.160*		0.0575	1
March	*6.577*	*-4.637*	*-3.037*	*-2.891*		0.465
April	*2.922*	*1.789*	*0.280*	*0.394*	*-2.156*	

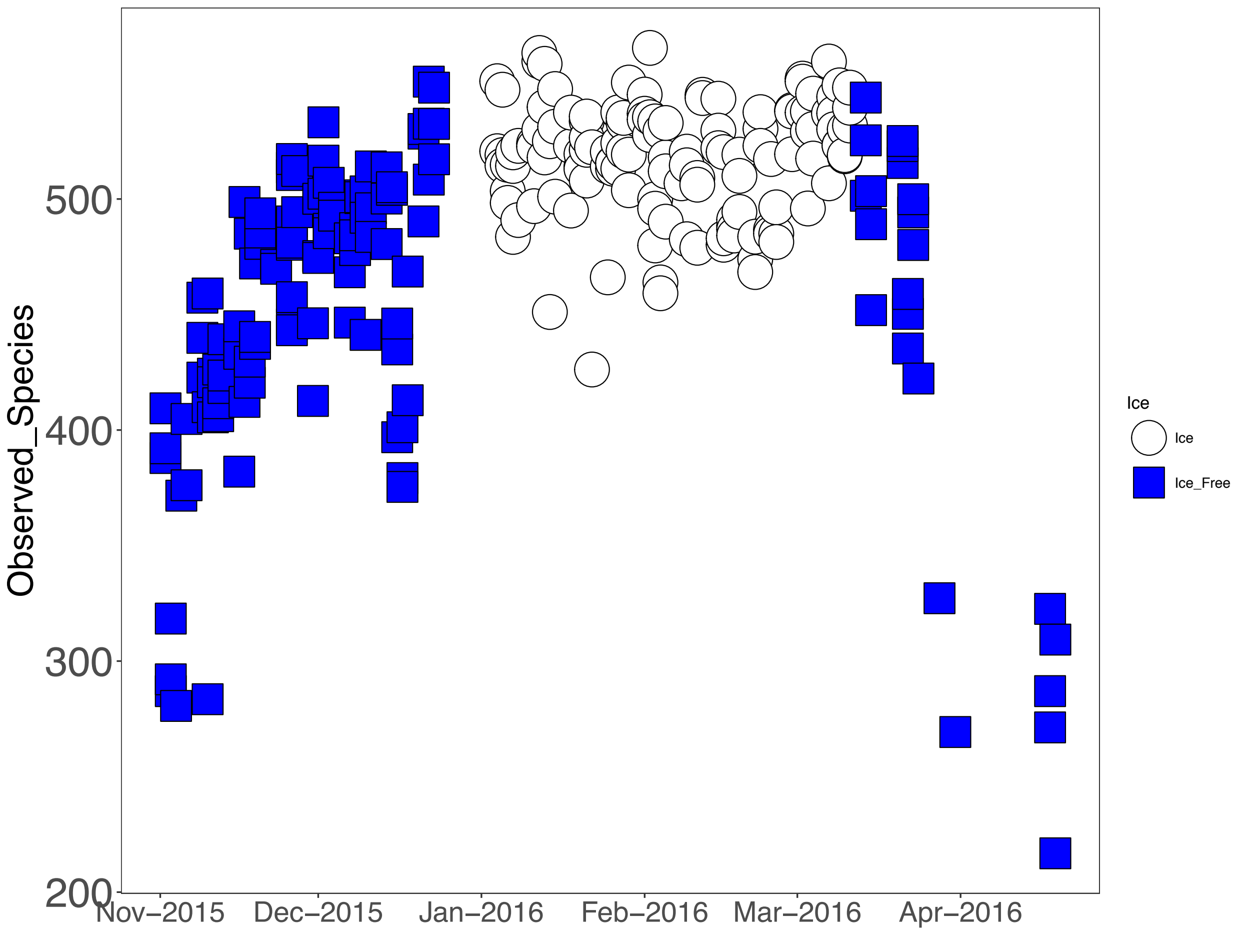

Supplemental Figure 3: Observed species alpha diversity metric across the time series. Samples from ice-free times are colored in blue and samples from ice-covered times are white. Kruskal-Wallis test for significance of ice cover on Observed Species: p-value = <0.00001, chi squared = 94.206, degrees of freedom = 1)

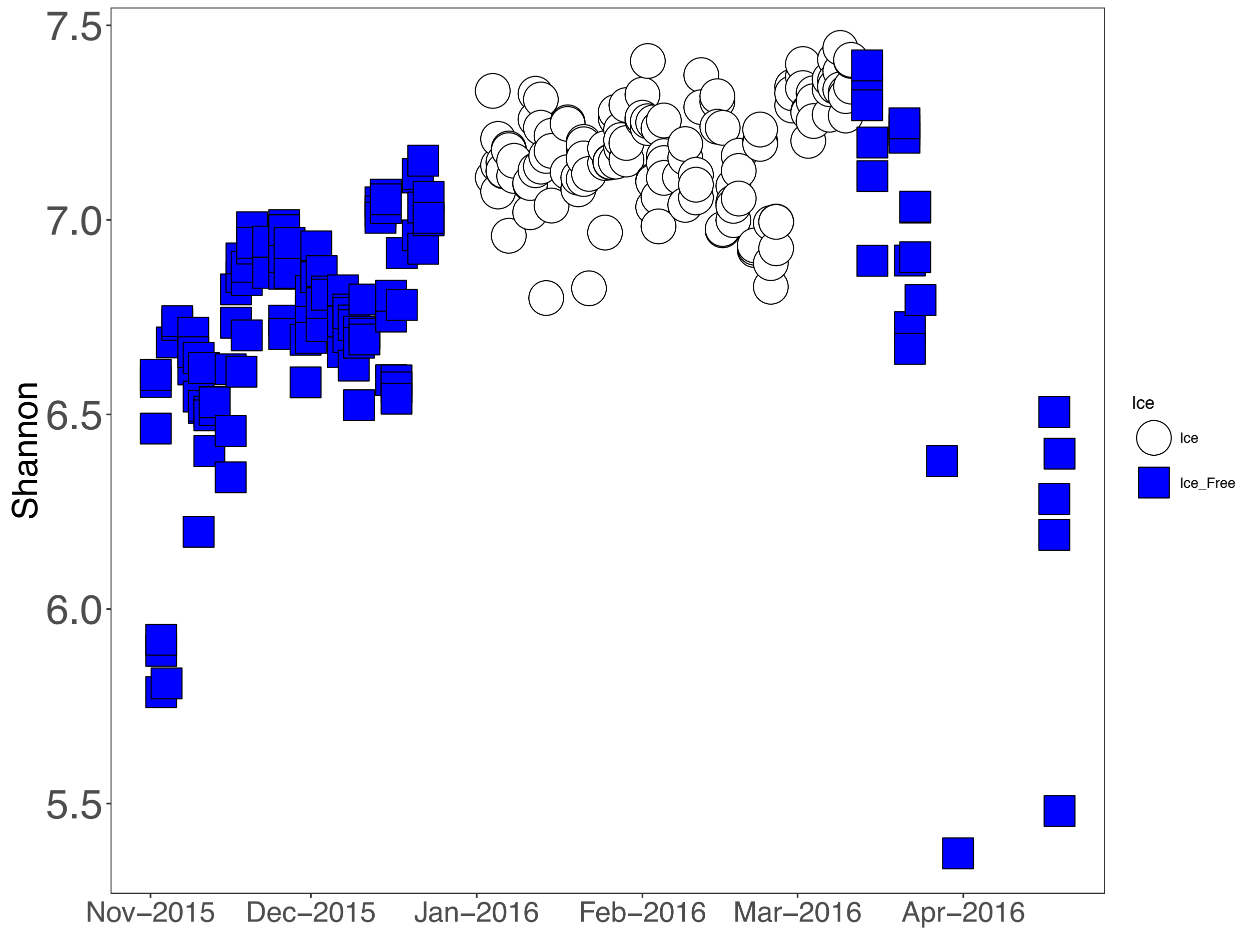

Supplemental Figure 4: Shannon diversity metric across the time series. Samples from ice-free times are colored in blue and samples from ice-covered times are white. Kruskal-Wallis test for significance of ice cover on Shannon diversity: p-value = <0.00001, chi squared = 139.82, degrees of freedom = 1)

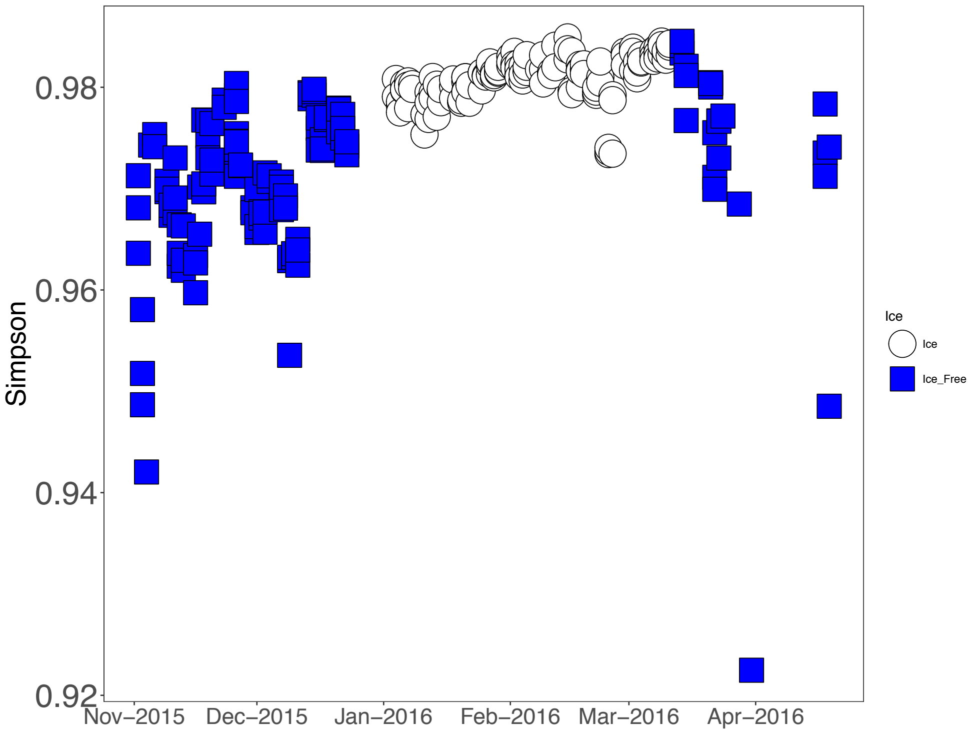

Supplemental Figure 5: Simpson diversity metric across the time series. Samples from ice-free times are colored in blue and samples from ice-covered times are white. Kruskal-Wallis test for significance of ice cover on Shannon diversity: p-value = <0.00001, chi squared = 144.96, degrees of freedom = 1)

Supplemental Table 3: Dunn test comparing Faith's Phylogenetic Diversity for each month. P-values of adjusted for multiple comparisons using Bonferroni correction and shown in the upper triangle and significant comparisons are shown in bold. Z stats are shown in the lower triangle in italic.

Faith's Phylogenetic	November	December	January	February	March	April
November		**0.01988**	**<0.00001**	**<0.00001**	**0.000015**	1
December	*3.210*		**<0.00001**	**<0.00001**	1	**0.078**
January	*9.465*	*-6.006*		1	**0.00127**	**<0.00001**
February	*8.954*	*-5.523*	*-0.469*		**0.00759**	**<0.00001**
March	*4.898*	*-1.780*	*3.930*	*3.477*		**0.00548**
April	*-1.450*	*-2.793*	*-5.333*	*-5.137*	-3.563	

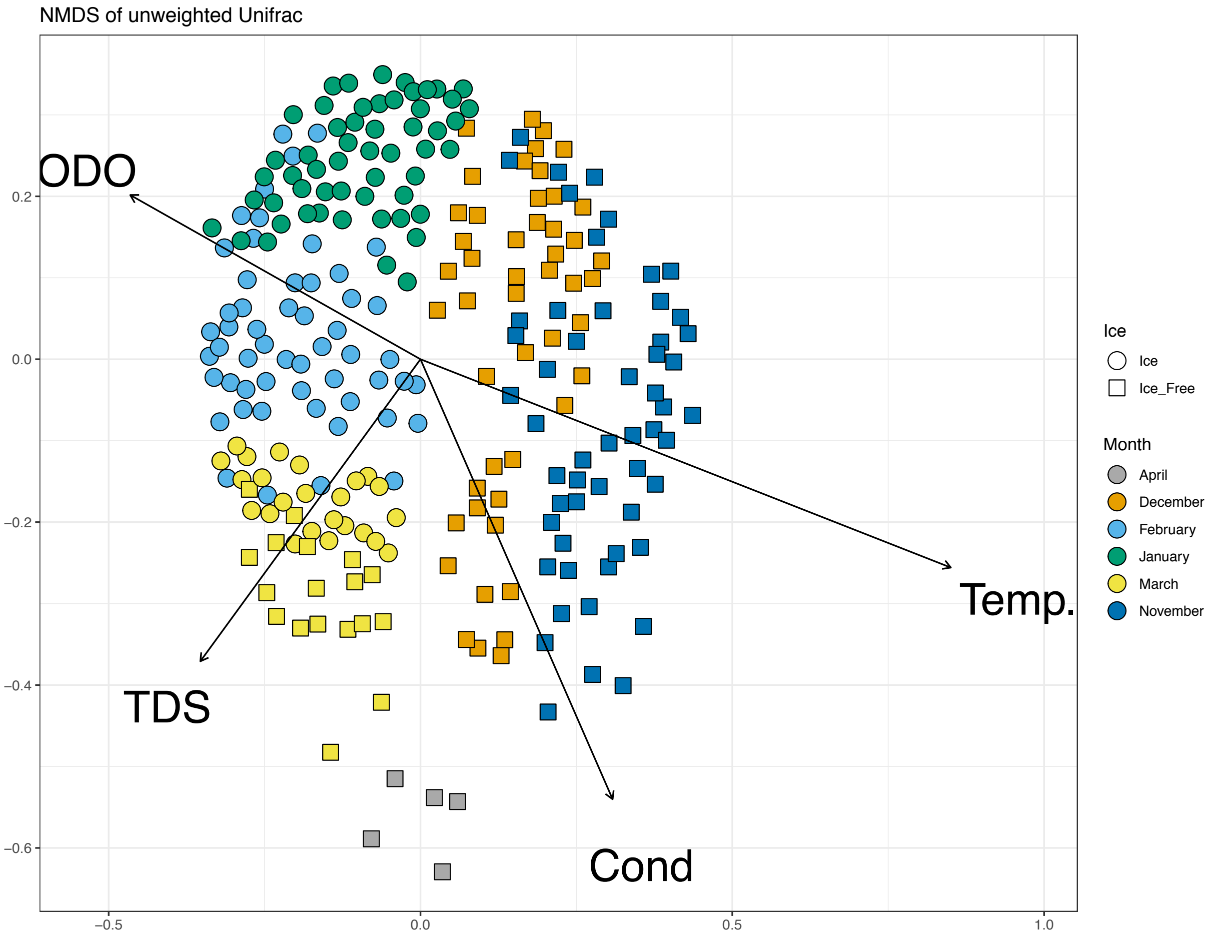

Supplementary Figure 6: Non-metric multidimensional scaling of unweighted unifrac distances. Stress for this plot is 0.2334. Environmental factors were fit to the NMDS and factors that significantly fit the data are shown as vectors.

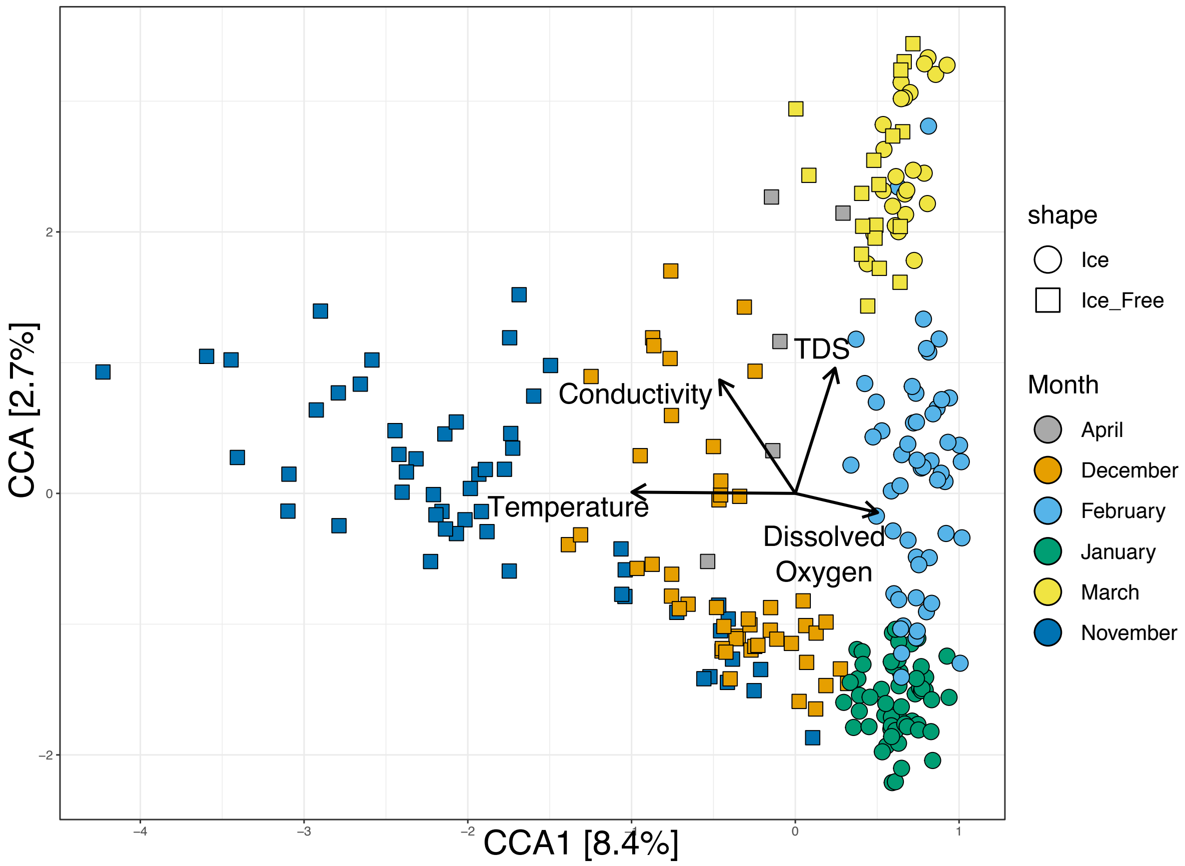

Supplementary Figure 7: CCA analysis of weighted unifrac distances.

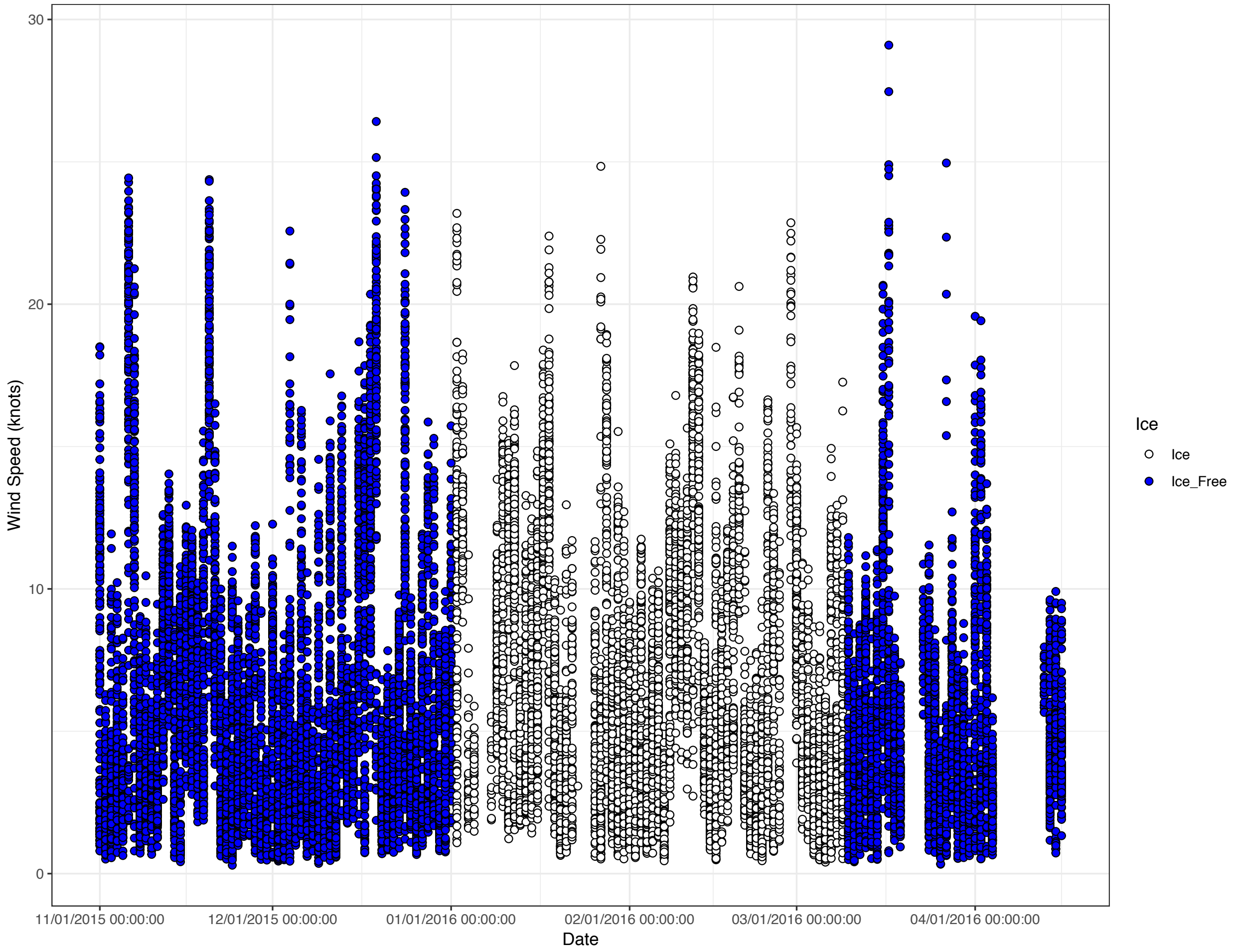

Supplementary Figure 8: Wind Speeds measured that GLRCMET weather station located at the Michigan Tech Great Lakes Research Center (47.120138, 552871). (http://uglos.mtu.edu/station_page.php?station=GLRCMET). Wind speeds measured during times of ice cover are shown as white circles and times of high wind are shown as blue circles.

Supplemental Table 4: qPCR data for archaeal 16S rRNA gene copy numbers for determination of bacterial abundance.

Date	Copies_Archaea	log_archaeal_copies	Ice	Month
11/2/15	109246.1949	5.038406319	Ice_Free	November
11/2/15	101986.0692	5.008540853	Ice_Free	November
11/2/15	97462.85977	4.98883915	Ice_Free	November
11/2/15	68546.14317	4.835983024	Ice_Free	November
11/2/15	51825.79809	4.714545999	Ice_Free	November
11/2/15	29521.66711	4.470140879	Ice_Free	November
11/3/15	391318.5314	5.592530415	Ice_Free	November
11/3/15	354412.1335	5.549508582	Ice_Free	November
11/3/15	194848.9632	5.289698099	Ice_Free	November

11/3/15	124765.6855	5.096095157	Ice_Free	November
11/3/15	73451.9452	4.866003302	Ice_Free	November
11/3/15	23620.60344	4.373290988	Ice_Free	November
11/4/15	302551.3032	5.480799028	Ice_Free	November
11/4/15	27090.29529	4.432813739	Ice_Free	November
11/4/15	19529.31272	4.29068696	Ice_Free	November
11/4/15	16229.484	4.210304712	Ice_Free	November
11/4/15	10787.06296	4.032903214	Ice_Free	November
11/4/15	1328.192983	3.123261181	Ice_Free	November
11/5/15	255423103.3	8.407260177	Ice_Free	November
11/5/15	1282.360713	3.108010204	Ice_Free	November
11/5/15	92.94243221	1.968214033	Ice_Free	November
11/5/15	4.557442058	0.658721156	Ice_Free	November
11/6/15	350856.8432	5.545129952	Ice_Free	November
11/6/15	247517.6078	5.393606099	Ice_Free	November
11/6/15	197120.9763	5.294732841	Ice_Free	November
11/6/15	196520.0754	5.293406922	Ice_Free	November
11/6/15	19022.72939	4.27927283	Ice_Free	November
11/7/15	384839.0194	5.585279099	Ice_Free	November
11/8/15	12379.31098	4.092696473	Ice_Free	November
11/9/15	672481.6396	5.827680432	Ice_Free	November
11/9/15	535183.5971	5.728502794	Ice_Free	November
11/9/15	256164.1268	5.408518311	Ice_Free	November
11/9/15	227130.9931	5.3562764	Ice_Free	November
11/10/15	41477.82159	4.617815939	Ice_Free	November
11/10/15	6668.890094	3.82405356	Ice_Free	November
11/11/15	179345.6379	5.253690818	Ice_Free	November
11/11/15	147539.313	5.168907757	Ice_Free	November
11/11/15	139827.9754	5.145594069	Ice_Free	November
11/11/15	128801.7353	5.109921714	Ice_Free	November
11/11/15	57261.42379	4.757862142	Ice_Free	November
11/11/15	29087.98996	4.463713712	Ice_Free	November
11/11/15	15482.16741	4.189831759	Ice_Free	November
11/12/15	1119924.262	6.049188653	Ice_Free	November
11/12/15	276259.9551	5.441317937	Ice_Free	November
11/12/15	257387.9566	5.410588222	Ice_Free	November
11/12/15	222364.3657	5.347065192	Ice_Free	November
11/12/15	221943.0092	5.34624147	Ice_Free	November
11/12/15	54945.61134	4.73993301	Ice_Free	November

11/12/15	12969.53952	4.112924557	Ice_Free	November
11/13/15	2291966.865	6.360208335	Ice_Free	November
11/13/15	657981.6442	5.818213778	Ice_Free	November
11/13/15	246892.5747	5.392508029	Ice_Free	November
11/13/15	124630.5801	5.095624616	Ice_Free	November
11/13/15	43537.93568	4.638867833	Ice_Free	November
11/13/15	38116.86574	4.581117182	Ice_Free	November
11/13/15	31765.13802	4.501950747	Ice_Free	November
11/13/15	249.0974745	2.396369324	Ice_Free	November
11/16/15	212681.0049	5.327728704	Ice_Free	November
11/16/15	113294.1828	5.054207611	Ice_Free	November
11/16/15	80099.59894	4.903630342	Ice_Free	November
11/16/15	62039.65807	4.792669396	Ice_Free	November
11/16/15	15050.50953	4.177551203	Ice_Free	November
11/16/15	348.7633218	2.542530805	Ice_Free	November
11/17/15	717243519.4	8.855666633	Ice_Free	November
11/17/15	796282.7804	5.901067324	Ice_Free	November
11/17/15	233996.6343	5.369209611	Ice_Free	November
11/17/15	165865.1533	5.219755154	Ice_Free	November
11/17/15	20013.10537	4.301314482	Ice_Free	November
11/17/15	18973.6307	4.278150443	Ice_Free	November
11/18/15	847246.3796	5.928009722	Ice_Free	November
11/18/15	154159.2549	5.187969603	Ice_Free	November
11/18/15	86850.95722	4.938774609	Ice_Free	November
11/18/15	49761.37889	4.696892406	Ice_Free	November
11/18/15	11594.13409	4.064238319	Ice_Free	November
11/18/15	7512.632187	3.875792127	Ice_Free	November
11/19/15	595053.3209	5.774555883	Ice_Free	November
11/19/15	190402.6469	5.279672981	Ice_Free	November
11/19/15	108012.3797	5.033473534	Ice_Free	November
11/19/15	107362.8931	5.030854206	Ice_Free	November
11/19/15	80604.34504	4.906358453	Ice_Free	November
11/19/15	64978.16019	4.81276741	Ice_Free	November
11/20/15	464788.6457	5.66725551	Ice_Free	November
11/20/15	303790.4476	5.482574114	Ice_Free	November
11/20/15	177130.5656	5.248293509	Ice_Free	November
11/20/15	100714.0766	5.003090175	Ice_Free	November
11/20/15	37298.69317	4.571693616	Ice_Free	November
11/20/15	36741.27377	4.565154209	Ice_Free	November

11/23/15	1009285.869	6.004014193	Ice_Free	November
11/23/15	219141.5197	5.340724669	Ice_Free	November
11/23/15	113467.6009	5.054871872	Ice_Free	November
11/23/15	32048.87309	4.505812763	Ice_Free	November
11/23/15	32041.72768	4.505715925	Ice_Free	November
11/23/15	20796.64353	4.317993248	Ice_Free	November
11/24/15	901659.4911	5.955042559	Ice_Free	November
11/24/15	818474.0099	5.913004893	Ice_Free	November
11/24/15	158341.3974	5.199594473	Ice_Free	November
11/24/15	69701.96221	4.843245004	Ice_Free	November
11/24/15	62317.30717	4.794608679	Ice_Free	November
11/24/15	34470.44706	4.537446916	Ice_Free	November
11/25/15	688165.3403	5.837692795	Ice_Free	November
11/25/15	138937.3566	5.142819032	Ice_Free	November
11/25/15	115120.1736	5.061151436	Ice_Free	November
11/25/15	62454.70502	4.795565161	Ice_Free	November
11/25/15	36481.08548	4.562067752	Ice_Free	November
11/25/15	24853.96539	4.395395689	Ice_Free	November
11/25/15	21833.85702	4.339130462	Ice_Free	November
11/26/15	1250721.921	6.097160762	Ice_Free	November
11/26/15	324328.9561	5.510985724	Ice_Free	November
11/26/15	243175.8596	5.38592046	Ice_Free	November
11/26/15	68964.66398	4.838626625	Ice_Free	November
11/26/15	716.9544498	2.855491565	Ice_Free	November
11/27/15	126035.0325	5.100491278	Ice_Free	November
11/27/15	68115.49377	4.833245909	Ice_Free	November
11/27/15	45282.87546	4.655933997	Ice_Free	November
11/27/15	16683.16872	4.222278542	Ice_Free	November
11/27/15	2710.179617	3.432998075	Ice_Free	November
11/30/15	567307.3285	5.753818394	Ice_Free	November
11/30/15	463891.9724	5.666416857	Ice_Free	November
11/30/15	42204.15937	4.625355254	Ice_Free	November
11/30/15	36531.50403	4.562667553	Ice_Free	November
11/30/15	3.006739724	0.478095835	Ice_Free	November
12/1/15	50712978.53	7.705119119	Ice_Free	December
12/1/15	892832.8316	5.950770152	Ice_Free	December
12/1/15	521644.1216	5.717374318	Ice_Free	December
12/1/15	305889.7803	5.485564968	Ice_Free	December
12/1/15	291477.0503	5.464604366	Ice_Free	December

12/1/15	185742.248	5.268910697	Ice_Free	December
12/2/15	1293406.83	6.11173515	Ice_Free	December
12/2/15	902188.7523	5.955297408	Ice_Free	December
12/2/15	211909.2206	5.326149854	Ice_Free	December
12/2/15	198107.6531	5.296901253	Ice_Free	December
12/2/15	33273.84123	4.52210294	Ice_Free	December
12/2/15	29134.13989	4.464402201	Ice_Free	December
12/3/15	412300703.2	8.615214076	Ice_Free	December
12/3/15	275660.3734	5.44037434	Ice_Free	December
12/3/15	80642.05001	4.906561559	Ice_Free	December
12/3/15	35070.66474	4.544943998	Ice_Free	December
12/3/15	31269.89439	4.495126414	Ice_Free	December
12/3/15	19277.1591	4.285043032	Ice_Free	December
12/3/15	11199.98197	4.049217324	Ice_Free	December
12/4/15	454795.5004	5.657816159	Ice_Free	December
12/4/15	209003.4976	5.320153554	Ice_Free	December
12/4/15	4714.607854	3.673445575	Ice_Free	December
12/4/15	525.931142	2.720928887	Ice_Free	December
12/7/15	380106.7975	5.579905636	Ice_Free	December
12/7/15	350048.9019	5.54412872	Ice_Free	December
12/7/15	248190.2708	5.394784753	Ice_Free	December
12/7/15	200144.7745	5.301344256	Ice_Free	December
12/7/15	189728.8522	5.278133379	Ice_Free	December
12/8/15	1059397.234	6.025058835	Ice_Free	December
12/8/15	106342.3886	5.026706411	Ice_Free	December
12/8/15	35124.58497	4.545611201	Ice_Free	December
12/8/15	27087.95093	4.432776154	Ice_Free	December
12/8/15	18553.92902	4.268435891	Ice_Free	December
12/8/15	2175.471546	3.337553407	Ice_Free	December
12/9/15	660697.2744	5.820002515	Ice_Free	December
12/9/15	261047.3736	5.416719328	Ice_Free	December
12/9/15	123171.584	5.090510527	Ice_Free	December
12/9/15	69731.13533	4.843426736	Ice_Free	December
12/9/15	13.64898165	1.13510025	Ice_Free	December
12/10/15	385953.5252	5.586535012	Ice_Free	December
12/10/15	88489.88503	4.946893631	Ice_Free	December
12/10/15	56539.08219	4.752348754	Ice_Free	December
12/10/15	12011.46528	4.07959599	Ice_Free	December
12/10/15	1257.258086	3.099424437	Ice_Free	December

12/11/15	65753731.21	7.817920402	Ice_Free	December
12/11/15	893321.4631	5.951007769	Ice_Free	December
12/11/15	85636.48178	4.932658817	Ice_Free	December
12/11/15	82086.36876	4.914271044	Ice_Free	December
12/11/15	38643.53032	4.587076795	Ice_Free	December
12/14/15	148735.2928	5.172414033	Ice_Free	December
12/14/15	121250.8842	5.083684914	Ice_Free	December
12/14/15	50728.05782	4.705248235	Ice_Free	December
12/14/15	48699.84985	4.687527622	Ice_Free	December
12/14/15	11344.4643	4.054783993	Ice_Free	December
12/14/15	9.049953072	0.956646327	Ice_Free	December
12/15/15	868226.2932	5.938632934	Ice_Free	December
12/15/15	79588.40513	4.900849802	Ice_Free	December
12/15/15	45257.93327	4.655694718	Ice_Free	December
12/15/15	24585.29342	4.390675396	Ice_Free	December
12/15/15	21457.71695	4.331583512	Ice_Free	December
12/16/15	1341451.07	6.127574836	Ice_Free	December
12/16/15	55926.19032	4.747615236	Ice_Free	December
12/16/15	53859.45107	4.731261923	Ice_Free	December
12/21/15	7364175.857	6.867124151	Ice_Free	December
12/21/15	836755.7737	5.922598718	Ice_Free	December
12/21/15	341158.4164	5.53295609	Ice_Free	December
12/21/15	81306.52169	4.910125382	Ice_Free	December
12/21/15	17930.32228	4.253588096	Ice_Free	December
1/4/16	2928497.093	6.466644797	Ice	January
1/4/16	1343934.57	6.128378125	Ice	January
1/4/16	1204403.587	6.08077204	Ice	January
1/4/16	21623.73058	4.334930622	Ice	January
1/7/16	1309501.661	6.117106054	Ice	January
1/7/16	1267689.435	6.103012871	Ice	January
1/7/16	1054405.683	6.023007738	Ice	January
1/7/16	56763.68208	4.754070559	Ice	January
1/12/16	1488320.276	6.172696398	Ice	January
1/12/16	879277.2674	5.944125845	Ice	January
1/12/16	660955.624	5.820172302	Ice	January
1/12/16	198864.9157	5.29855817	Ice	January
1/12/16	1951.317099	3.29032785	Ice	January
1/12/16	350.5097591	2.544700114	Ice	January
1/15/16	1297292.743	6.113037989	Ice	January

1/15/16	654100.3204	5.815644362	Ice	January
1/15/16	610613.0728	5.785766098	Ice	January
1/15/16	258021.3107	5.411655577	Ice	January
1/15/16	58066.76028	4.763927596	Ice	January
1/15/16	25463.35988	4.405915708	Ice	January
1/20/16	144605763.5	8.160185603	Ice	January
1/20/16	6541246.699	6.815660529	Ice	January
1/20/16	565576.3475	5.752491239	Ice	January
1/20/16	447046.1432	5.650352352	Ice	January
1/20/16	204749.2127	5.31122224	Ice	January
1/20/16	105886.2593	5.024839606	Ice	January
1/20/16	31154.46564	4.493520307	Ice	January
1/20/16	6238.217848	3.795060537	Ice	January
1/21/16	578005105.7	8.761931675	Ice	January
1/21/16	877870.562	5.943430486	Ice	January
1/21/16	343658.7767	5.53612744	Ice	January
1/21/16	236876.8072	5.374522541	Ice	January
1/21/16	221038.3674	5.344467664	Ice	January
1/21/16	178840.7647	5.252466518	Ice	January
1/21/16	88533.88761	4.947109535	Ice	January
1/21/16	42481.54459	4.628200299	Ice	January
1/21/16	35185.67623	4.546365902	Ice	January
1/21/16	14648.96307	4.165806884	Ice	January
1/21/16	1913.450206	3.281817165	Ice	January
1/22/16	686263.7532	5.836491061	Ice	January
1/22/16	620811.662	5.792959867	Ice	January
1/22/16	307516.9668	5.487869082	Ice	January
1/22/16	263911.5734	5.421458436	Ice	January
1/22/16	214959.4136	5.332356469	Ice	January
1/22/16	191932.4901	5.283148498	Ice	January
1/22/16	145.2790694	2.162203049	Ice	January
1/25/16	612929.6689	5.787410644	Ice	January
1/25/16	581499.7547	5.764549536	Ice	January
1/25/16	563163.7243	5.750634672	Ice	January
1/25/16	494283.5069	5.693976119	Ice	January
1/25/16	478955.3547	5.680295033	Ice	January
1/25/16	263940.6095	5.421506215	Ice	January
1/25/16	186177.5398	5.269927287	Ice	January
1/25/16	98962.02428	4.99546857	Ice	January

1/25/16	59675.90886	4.775799042	Ice	January
1/25/16	35995.27941	4.556245549	Ice	January
1/25/16	15671.12874	4.195100278	Ice	January
1/25/16	11269.23002	4.051894244	Ice	January
1/25/16	3093.785542	3.490490206	Ice	January
1/26/16	401731.6578	5.603936057	Ice	January
1/26/16	291934.6813	5.465285691	Ice	January
1/26/16	173726.4765	5.239866011	Ice	January
1/26/16	19798.16137	4.29662486	Ice	January
1/27/16	568072.4008	5.75440369	Ice	January
1/27/16	547508.9598	5.738391231	Ice	January
1/27/16	531106.9257	5.725181965	Ice	January
1/27/16	346313.8471	5.539469857	Ice	January
1/27/16	315321.0379	5.498752947	Ice	January
1/27/16	310350.9698	5.491853107	Ice	January
1/27/16	253247.6769	5.40354547	Ice	January
1/27/16	162742.7887	5.211501754	Ice	January
1/27/16	123647.749	5.092186214	Ice	January
1/27/16	120688.3602	5.081665387	Ice	January
1/27/16	75875.36518	4.880100794	Ice	January
1/27/16	31385.01727	4.496722372	Ice	January
1/28/16	508264.1145	5.706089448	Ice	January
1/28/16	482881.9129	5.683840939	Ice	January
1/28/16	421953.7151	5.625264815	Ice	January
1/28/16	200593.3462	5.302316523	Ice	January
1/28/16	187218.0941	5.27234782	Ice	January
1/28/16	19151.48001	4.282202342	Ice	January
1/28/16	8924.147743	3.950566752	Ice	January
1/28/16	2441.699537	3.387692221	Ice	January
1/29/16	528787.3191	5.723281032	Ice	January
1/29/16	340216.7059	5.531755635	Ice	January
1/29/16	297146.403	5.472970477	Ice	January
1/29/16	263469.6867	5.420730655	Ice	January
1/29/16	250512.0065	5.398828545	Ice	January
1/29/16	223449.5423	5.349179469	Ice	January
1/29/16	223038.8136	5.348380446	Ice	January
1/29/16	124012.2927	5.093464737	Ice	January
1/29/16	75793.20178	4.879630254	Ice	January
1/29/16	6589.319151	3.818840543	Ice	January

2/1/16	1637830.931	6.214269069	Ice	February
2/1/16	843657.0756	5.926165953	Ice	February
2/1/16	671240.9853	5.826878466	Ice	February
2/1/16	316432.7736	5.500281458	Ice	February
2/1/16	250023.7798	5.397981316	Ice	February
2/1/16	233098.9762	5.367540366	Ice	February
2/1/16	164926.9316	5.217291579	Ice	February
2/1/16	149250.896	5.173916947	Ice	February
2/1/16	133848.9684	5.126615028	Ice	February
2/1/16	82731.21263	4.91766939	Ice	February
2/2/16	363708.4534	5.560753395	Ice	February
2/2/16	331309.2607	5.520233575	Ice	February
2/2/16	291424.6892	5.464526342	Ice	February
2/2/16	177202.4392	5.248469696	Ice	February
2/2/16	79590.75051	4.9008626	Ice	February
2/2/16	46877.87084	4.670967878	Ice	February
2/3/16	686203.7604	5.836453094	Ice	February
2/3/16	657168.4238	5.817676688	Ice	February
2/3/16	296560.5896	5.472113436	Ice	February
2/3/16	289897.4896	5.462244455	Ice	February
2/3/16	286044.8626	5.456434152	Ice	February
2/3/16	222239.4842	5.34682122	Ice	February
2/3/16	120397.5515	5.080617655	Ice	February
2/3/16	66215.78893	4.820961558	Ice	February
2/4/16	719107.5432	5.856793844	Ice	February
2/4/16	489819.9406	5.690036461	Ice	February
2/4/16	105420.1772	5.022923742	Ice	February
2/4/16	100340.9238	5.001478095	Ice	February
2/4/16	93033.01356	4.968637089	Ice	February
2/5/16	687714.6132	5.837408253	Ice	February
2/5/16	1177.097195	3.070812325	Ice	February
2/8/16	465742.9575	5.668146297	Ice	February
2/9/16	441519.9247	5.644950307	Ice	February
2/9/16	398049.9081	5.599937528	Ice	February
2/9/16	170765.9787	5.232401352	Ice	February
2/9/16	42103.79265	4.624321218	Ice	February
2/12/16	376006.6599	5.575195537	Ice	February
2/12/16	293401.418	5.467462208	Ice	February
2/12/16	267921.7908	5.428008037	Ice	February

2/12/16	81954.24678	4.913571463	Ice	February
2/12/16	11901.94498	4.075617938	Ice	February
2/18/16	314316.3131	5.497366922	Ice	February
2/18/16	154516.7366	5.188975527	Ice	February
2/19/16	217078.9857	5.336617784	Ice	February
2/19/16	195757.1471	5.291717627	Ice	February
2/22/16	1815422.365	6.258977681	Ice	February
2/22/16	829444.7575	5.918787467	Ice	February
2/22/16	640436.809	5.806476285	Ice	February
2/22/16	327509.2542	5.515223576	Ice	February
2/22/16	91016.126	4.959118346	Ice	February
2/22/16	2763.042755	3.441387605	Ice	February
2/25/16	572992.9054	5.758149245	Ice	February
2/25/16	131813.6795	5.119960483	Ice	February
2/25/16	129619.6157	5.11267073	Ice	February
2/25/16	44342.14823	4.646816729	Ice	February
2/26/16	14161.37323	4.151105369	Ice	February
2/26/16	6650.861324	3.822877893	Ice	February
2/26/16	1623.899149	3.210559054	Ice	February
2/26/16	1451.399835	3.161787069	Ice	February
2/29/16	600196.9154	5.778293759	Ice	February
2/29/16	305175.2262	5.484549275	Ice	February
2/29/16	106688.9184	5.028119312	Ice	February
2/29/16	1228055.182	6.089217882	Ice	February
3/2/16	1003089.842	6.001339832	Ice	March
3/2/16	548246.2549	5.738975674	Ice	March
3/2/16	350317.2414	5.544461512	Ice	March
3/2/16	291215.7929	5.464214923	Ice	March
3/2/16	8523.987218	3.93064279	Ice	March
3/2/16	3633.907002	3.560373809	Ice	March
3/3/16	1517803.729	6.181215615	Ice	March
3/3/16	482133.5304	5.683167336	Ice	March
3/3/16	478662.8114	5.680029687	Ice	March
3/3/16	273643.9974	5.437185926	Ice	March
3/3/16	224890.518	5.351971145	Ice	March
3/3/16	169119.872	5.228194641	Ice	March
3/4/16	27517956.66	7.439616182	Ice	March
3/4/16	777008.4975	5.890425768	Ice	March
3/4/16	650092.448	5.812975121	Ice	March

3/4/16	1493.304084	3.174148253	Ice	March
3/7/16	636416.3779	5.803741347	Ice	March
3/7/16	614533.2728	5.788545402	Ice	March
3/7/16	502928.2711	5.701506049	Ice	March
3/7/16	299067.921	5.475769832	Ice	March
3/7/16	65167.17113	4.814028869	Ice	March
3/7/16	736.9906563	2.867461982	Ice	March
3/8/16	726833.3295	5.861434834	Ice	March
3/8/16	500244.5375	5.699182355	Ice	March
3/8/16	379582.5702	5.579306262	Ice	March
3/8/16	111795.4758	5.048424229	Ice	March
3/8/16	46016.77889	4.662916215	Ice	March
3/8/16	2300.303729	3.361785183	Ice	March
3/9/16	805369299.9	8.905995071	Ice	March
3/9/16	1024977.623	6.010714384	Ice	March
3/9/16	703409.3314	5.847208125	Ice	March
3/9/16	513690.3752	5.710701429	Ice	March
3/9/16	195.7670138	2.291739516	Ice	March
3/10/16	802653.1411	5.90452791	Ice_Free	March
3/10/16	371272.5271	5.569692814	Ice_Free	March
3/10/16	360959.3791	5.557458331	Ice_Free	March
3/10/16	328443.5354	5.516460718	Ice_Free	March
3/10/16	90842.04481	4.958286901	Ice_Free	March
3/11/16	311130.5417	5.492942645	Ice_Free	March
3/11/16	222892.5111	5.348095477	Ice_Free	March
3/11/16	181112.2856	5.257947911	Ice_Free	March
3/11/16	103711.3733	5.015826385	Ice_Free	March
3/11/16	78005.29975	4.89212411	Ice_Free	March
3/11/16	18725.56328	4.27243489	Ice_Free	March
3/14/16	1823326.888	6.260864536	Ice_Free	March
3/14/16	1505581.482	6.177704264	Ice_Free	March
3/14/16	673732.0036	5.828487178	Ice_Free	March
3/14/16	386395.8279	5.587032428	Ice_Free	March
3/14/16	315233.0855	5.498631793	Ice_Free	March
3/15/16	1146398.948	6.059335779	Ice_Free	March
3/15/16	877324.884	5.943160448	Ice_Free	March
3/15/16	773937.9462	5.888706141	Ice_Free	March
3/15/16	563308.678	5.750746442	Ice_Free	March
3/15/16	9783.341138	3.990487197	Ice_Free	March

3/21/16	3710234.861	6.569401402	Ice_Free	March
3/21/16	1274119.878	6.105210291	Ice_Free	March
3/21/16	1114757.022	6.047180217	Ice_Free	March
3/21/16	513833.1941	5.710822157	Ice_Free	March
3/21/16	369519.5766	5.567637452	Ice_Free	March
3/22/16	8889258.768	6.948865549	Ice_Free	March
3/22/16	823663.9706	5.915750069	Ice_Free	March
3/22/16	451628.4805	5.654781322	Ice_Free	March
3/22/16	451250.6678	5.654417858	Ice_Free	March
3/22/16	73759.45153	4.867817679	Ice_Free	March
3/22/16	42057.86908	4.623847265	Ice_Free	March
3/23/16	2216917.621	6.345749555	Ice_Free	March
3/23/16	1228482.287	6.089368899	Ice_Free	March
3/23/16	627610.8491	5.797690442	Ice_Free	March
3/23/16	122504.8071	5.088153131	Ice_Free	March
3/23/16	33444.2382	4.524321308	Ice_Free	March
3/24/16	2892455.203	6.461266642	Ice_Free	March
3/24/16	595526.9638	5.77490143	Ice_Free	March
3/24/16	581862.5788	5.764820427	Ice_Free	March
3/24/16	165.0345884	2.217574974	Ice_Free	March
3/24/16	119.5600081	2.077585936	Ice_Free	March
3/28/16	1431610.137	6.155824765	Ice_Free	March
3/28/16	1146120.837	6.059230408	Ice_Free	March
3/28/16	770884.0725	5.886989073	Ice_Free	March
3/28/16	583123.0769	5.765760229	Ice_Free	March
3/28/16	404788.8689	5.607228562	Ice_Free	March
3/28/16	284000.7848	5.45331954	Ice_Free	March
3/29/16	6222802.401	6.793986011	Ice_Free	March
3/29/16	1721231.49	6.235839283	Ice_Free	March
3/29/16	1328975.607	6.12351701	Ice_Free	March
3/29/16	1079867.068	6.033370297	Ice_Free	March
3/29/16	867863.9096	5.938451628	Ice_Free	March
3/31/16	7949166.287	6.900321582	Ice_Free	March
3/31/16	2802209.891	6.447500662	Ice_Free	March
3/31/16	1087472.691	6.03641836	Ice_Free	March
3/31/16	365144.2708	5.562464491	Ice_Free	March
3/31/16	352532.0307	5.547198583	Ice_Free	March
3/31/16	92005.17049	4.963812234	Ice_Free	March
4/2/16	2525280.142	6.402309564	Ice_Free	April

4/2/16	459931.8505	5.662693486	Ice_Free	April
4/5/16	1001687.728	6.000732353	Ice_Free	April
4/5/16	887949.8535	5.94838844	Ice_Free	April
4/5/16	733390.4405	5.865335245	Ice_Free	April
4/5/16	79.45368568	1.900114048	Ice_Free	April
4/7/16	1001341.44	6.00058219	Ice_Free	April
4/7/16	743014.1593	5.87099709	Ice_Free	April
4/7/16	706753.1516	5.849267754	Ice_Free	April
4/7/16	672204.9867	5.82750173	Ice_Free	April
4/8/16	975692.8082	5.989313104	Ice_Free	April
4/8/16	864481.8716	5.93675589	Ice_Free	April
4/8/16	613521.1882	5.787829566	Ice_Free	April
4/8/16	68873.48159	4.838052037	Ice_Free	April
4/12/16	209246651.5	8.320658517	Ice_Free	April
4/12/16	14697423.11	7.167241197	Ice_Free	April
4/12/16	721617.4011	5.858306997	Ice_Free	April
4/12/16	161.5324277	2.20825972	Ice_Free	April
4/13/16	331174.8813	5.520057389	Ice_Free	April
4/13/16	210559.4882	5.323374816	Ice_Free	April
4/13/16	187151.3498	5.272192964	Ice_Free	April
4/13/16	115907.4074	5.064111192	Ice_Free	April
4/13/16	10629.86402	4.026527709	Ice_Free	April
4/13/16	4077.784925	3.610424316	Ice_Free	April
4/14/16	496943.0707	5.696306639	Ice_Free	April
4/14/16	366113.2609	5.56361546	Ice_Free	April
4/14/16	265177.8599	5.423537261	Ice_Free	April
4/14/16	4025.45469	3.604814943	Ice_Free	April
4/14/16	2622.559155	3.418725293	Ice_Free	April
4/18/16	325075.2985	5.51198397	Ice_Free	April
4/18/16	300992.6989	5.478555961	Ice_Free	April
4/18/16	235193.0944	5.371424566	Ice_Free	April
4/18/16	139641.4402	5.145014319	Ice_Free	April
4/19/16	151489.6404	5.180382935	Ice_Free	April
4/19/16	107832.0671	5.032747931	Ice_Free	April
4/19/16	85493.35489	4.93193236	Ice_Free	April
4/19/16	73441.13085	4.865939356	Ice_Free	April
4/19/16	6369.637417	3.804114711	Ice_Free	April
4/20/16	172580.7805	5.236992429	Ice_Free	April
4/20/16	165337.9641	5.218372586	Ice_Free	April

4/20/16	140780.5748	5.148542734	Ice_Free	April
4/20/16	133409.2418	5.125185916	Ice_Free	April
4/20/16	91221.71822	4.960098248	Ice_Free	April
4/20/16	78337.47785	4.893969585	Ice_Free	April

Supplemental Table 5: qPCR data for bacterial 16S rRNA gene copy numbers for determination of bacterial abundance.

Date	Copies_Bacteria	log_bacterial_copies	Ice	Month
11/2/15	1.15E+08	8.06	Ice_Free	November
11/2/15	6.58E+06	6.82	Ice_Free	November
11/2/15	5.33E+06	6.73	Ice_Free	November
11/2/15	9.14E+04	4.96	Ice_Free	November
11/2/15	7.23E+02	2.86	Ice_Free	November
11/3/15	6.03E+06	6.78	Ice_Free	November
11/3/15	4.17E+06	6.62	Ice_Free	November
11/3/15	3.06E+06	6.49	Ice_Free	November
11/3/15	1.09E+06	6.04	Ice_Free	November
11/3/15	5.00E+05	5.70	Ice_Free	November
11/3/15	4.47E+05	5.65	Ice_Free	November
11/4/15	3.64E+09	9.56	Ice_Free	November
11/4/15	1.08E+07	7.03	Ice_Free	November
11/4/15	3.04E+06	6.48	Ice_Free	November
11/4/15	2.89E+06	6.46	Ice_Free	November
11/5/15	1.34E+04	4.13	Ice_Free	November
11/5/15	4.81E+03	3.68	Ice_Free	November
11/5/15	4.33E+03	3.64	Ice_Free	November
11/5/15	3.28E+03	3.52	Ice_Free	November
11/5/15	1.70E+03	3.23	Ice_Free	November
11/5/15	1.46E+03	3.16	Ice_Free	November
11/6/15	9.06E+06	6.96	Ice_Free	November
11/6/15	5.68E+06	6.75	Ice_Free	November
11/6/15	3.21E+06	6.51	Ice_Free	November
11/6/15	2.47E+06	6.39	Ice_Free	November
11/6/15	2.12E+06	6.33	Ice_Free	November
11/6/15	7.03E+04	4.85	Ice_Free	November
11/9/15	5.32E+09	9.73	Ice_Free	November
11/9/15	7.65E+07	7.88	Ice_Free	November
11/9/15	5.54E+06	6.74	Ice_Free	November

11/9/15	9.33E+05	5.97	Ice_Free	November
11/10/15	1.18E+07	7.07	Ice_Free	November
11/10/15	9.42E+06	6.97	Ice_Free	November
11/10/15	7.32E+06	6.86	Ice_Free	November
11/10/15	6.27E+06	6.80	Ice_Free	November
11/10/15	8.02E+05	5.90	Ice_Free	November
11/10/15	5.54E+05	5.74	Ice_Free	November
11/11/15	4.80E+06	6.68	Ice_Free	November
11/11/15	3.37E+06	6.53	Ice_Free	November
11/11/15	3.32E+06	6.52	Ice_Free	November
11/11/15	3.03E+06	6.48	Ice_Free	November
11/11/15	2.04E+04	4.31	Ice_Free	November
11/12/15	2.72E+06	6.43	Ice_Free	November
11/12/15	2.43E+06	6.39	Ice_Free	November
11/12/15	2.15E+06	6.33	Ice_Free	November
11/12/15	2.08E+06	6.32	Ice_Free	November
11/12/15	1.43E+06	6.16	Ice_Free	November
11/12/15	1.26E+06	6.10	Ice_Free	November
11/13/15	8.88E+06	6.95	Ice_Free	November
11/13/15	4.79E+06	6.68	Ice_Free	November
11/13/15	4.50E+06	6.65	Ice_Free	November
11/13/15	3.08E+06	6.49	Ice_Free	November
11/13/15	2.98E+06	6.47	Ice_Free	November
11/13/15	2.57E+06	6.41	Ice_Free	November
11/16/15	8.88E+06	6.95	Ice_Free	November
11/16/15	4.58E+06	6.66	Ice_Free	November
11/16/15	3.43E+06	6.54	Ice_Free	November
11/16/15	1.00E+06	6.00	Ice_Free	November
11/16/15	2.10E+05	5.32	Ice_Free	November
11/17/15	1.14E+10	10.10	Ice_Free	November
11/17/15	3.55E+04	4.55	Ice_Free	November
11/18/15	8.98E+06	6.95	Ice_Free	November
11/18/15	5.44E+06	6.74	Ice_Free	November
11/18/15	4.94E+06	6.69	Ice_Free	November
11/18/15	4.06E+06	6.61	Ice_Free	November
11/18/15	1.18E+06	6.07	Ice_Free	November
11/18/15	7.04E+05	5.85	Ice_Free	November
11/19/15	1.34E+07	7.13	Ice_Free	November
11/19/15	1.14E+07	7.06	Ice_Free	November

11/19/15	6.24E+06	6.80	Ice_Free	November
11/19/15	4.71E+06	6.67	Ice_Free	November
11/19/15	3.10E+06	6.49	Ice_Free	November
11/19/15	2.81E+06	6.45	Ice_Free	November
11/20/15	7.42E+07	7.87	Ice_Free	November
11/20/15	2.49E+07	7.40	Ice_Free	November
11/20/15	1.57E+07	7.20	Ice_Free	November
11/20/15	1.08E+07	7.03	Ice_Free	November
11/20/15	8.29E+06	6.92	Ice_Free	November
11/20/15	5.91E+06	6.77	Ice_Free	November
11/23/15	9.32E+06	6.97	Ice_Free	November
11/23/15	8.35E+06	6.92	Ice_Free	November
11/23/15	3.60E+05	5.56	Ice_Free	November
11/23/15	7.01E+04	4.85	Ice_Free	November
11/24/15	1.02E+07	7.01	Ice_Free	November
11/24/15	3.22E+06	6.51	Ice_Free	November
11/24/15	2.90E+06	6.46	Ice_Free	November
11/24/15	2.25E+06	6.35	Ice_Free	November
11/24/15	2.03E+06	6.31	Ice_Free	November
11/24/15	7.50E+04	4.88	Ice_Free	November
11/25/15	6.87E+06	6.84	Ice_Free	November
11/25/15	4.66E+06	6.67	Ice_Free	November
11/25/15	2.42E+06	6.38	Ice_Free	November
11/25/15	7.16E+04	4.85	Ice_Free	November
11/26/15	1.32E+09	9.12	Ice_Free	November
11/26/15	2.56E+07	7.41	Ice_Free	November
11/26/15	1.93E+07	7.29	Ice_Free	November
11/26/15	1.65E+07	7.22	Ice_Free	November
11/26/15	1.60E+07	7.20	Ice_Free	November
11/27/15	4.53E+07	7.66	Ice_Free	November
11/27/15	4.43E+07	7.65	Ice_Free	November
11/27/15	3.42E+07	7.53	Ice_Free	November
11/27/15	2.46E+07	7.39	Ice_Free	November
11/30/15	1.43E+10	10.20	Ice_Free	November
11/30/15	2.11E+06	6.32	Ice_Free	November
11/30/15	1.54E+06	6.19	Ice_Free	November
12/1/15	2.36E+07	7.37	Ice_Free	December
12/1/15	1.49E+07	7.17	Ice_Free	December
12/1/15	1.07E+07	7.03	Ice_Free	December

12/1/15	6.93E+06	6.84	Ice_Free	December
12/1/15	5.85E+06	6.77	Ice_Free	December
12/1/15	4.34E+06	6.64	Ice_Free	December
12/2/15	2.28E+08	8.36	Ice_Free	December
12/2/15	1.72E+07	7.24	Ice_Free	December
12/2/15	1.68E+07	7.23	Ice_Free	December
12/2/15	1.30E+07	7.11	Ice_Free	December
12/2/15	1.25E+07	7.10	Ice_Free	December
12/2/15	1.16E+07	7.06	Ice_Free	December
12/3/15	1.84E+07	7.26	Ice_Free	December
12/3/15	1.38E+07	7.14	Ice_Free	December
12/3/15	1.20E+07	7.08	Ice_Free	December
12/3/15	1.14E+07	7.06	Ice_Free	December
12/3/15	7.04E+06	6.85	Ice_Free	December
12/3/15	6.34E+06	6.80	Ice_Free	December
12/4/15	1.25E+06	6.10	Ice_Free	December
12/4/15	8.46E+05	5.93	Ice_Free	December
12/4/15	2.97E+05	5.47	Ice_Free	December
12/4/15	6.63E+04	4.82	Ice_Free	December
12/7/15	5.06E+07	7.70	Ice_Free	December
12/7/15	1.40E+07	7.15	Ice_Free	December
12/7/15	1.24E+07	7.09	Ice_Free	December
12/7/15	8.49E+06	6.93	Ice_Free	December
12/7/15	7.43E+06	6.87	Ice_Free	December
12/7/15	2.98E+06	6.47	Ice_Free	December
12/8/15	1.76E+07	7.25	Ice_Free	December
12/8/15	1.17E+07	7.07	Ice_Free	December
12/8/15	1.13E+07	7.05	Ice_Free	December
12/8/15	8.69E+06	6.94	Ice_Free	December
12/8/15	8.21E+06	6.91	Ice_Free	December
12/8/15	9.56E+05	5.98	Ice_Free	December
12/9/15	2.39E+07	7.38	Ice_Free	December
12/9/15	2.20E+07	7.34	Ice_Free	December
12/9/15	1.30E+07	7.11	Ice_Free	December
12/10/15	1.39E+07	7.14	Ice_Free	December
12/10/15	1.33E+07	7.12	Ice_Free	December
12/10/15	7.46E+06	6.87	Ice_Free	December
12/11/15	3.13E+07	7.50	Ice_Free	December
12/11/15	1.96E+07	7.29	Ice_Free	December

12/11/15	1.02E+07	7.01	Ice_Free	December
12/11/15	3.73E+06	6.57	Ice_Free	December
12/11/15	2.67E+04	4.43	Ice_Free	December
12/14/15	1.01E+07	7.00	Ice_Free	December
12/14/15	9.72E+06	6.99	Ice_Free	December
12/14/15	7.56E+06	6.88	Ice_Free	December
12/14/15	7.39E+06	6.87	Ice_Free	December
12/14/15	6.79E+06	6.83	Ice_Free	December
12/14/15	6.14E+06	6.79	Ice_Free	December
12/15/15	8.63E+07	7.94	Ice_Free	December
12/15/15	1.38E+07	7.14	Ice_Free	December
12/15/15	1.15E+07	7.06	Ice_Free	December
12/15/15	8.03E+06	6.90	Ice_Free	December
12/15/15	1.66E+06	6.22	Ice_Free	December
12/15/15	1.24E+06	6.09	Ice_Free	December
12/16/15	3.16E+06	6.50	Ice_Free	December
12/16/15	2.54E+06	6.40	Ice_Free	December
12/16/15	2.22E+06	6.35	Ice_Free	December
12/16/15	2.03E+06	6.31	Ice_Free	December
12/16/15	1.85E+06	6.27	Ice_Free	December
12/16/15	6.89E+04	4.84	Ice_Free	December
12/17/15	6.68E+06	6.82	Ice_Free	December
12/17/15	5.65E+06	6.75	Ice_Free	December
12/17/15	5.20E+06	6.72	Ice_Free	December
12/17/15	3.85E+06	6.59	Ice_Free	December
12/17/15	2.68E+06	6.43	Ice_Free	December
12/17/15	2.66E+06	6.42	Ice_Free	December
12/18/15	3.02E+08	8.48	Ice_Free	December
12/18/15	2.65E+08	8.42	Ice_Free	December
12/18/15	2.33E+08	8.37	Ice_Free	December
12/18/15	1.13E+06	6.05	Ice_Free	December
12/18/15	4.78E+05	5.68	Ice_Free	December
12/18/15	2.90E+05	5.46	Ice_Free	December
12/21/15	3.52E+07	7.55	Ice_Free	December
12/21/15	2.44E+07	7.39	Ice_Free	December
12/21/15	2.43E+07	7.39	Ice_Free	December
12/21/15	1.43E+07	7.16	Ice_Free	December
12/21/15	9.13E+06	6.96	Ice_Free	December
12/21/15	8.23E+06	6.92	Ice_Free	December

12/22/15	1.54E+07	7.19	Ice_Free	December
12/22/15	1.04E+07	7.02	Ice_Free	December
12/22/15	1.91E+06	6.28	Ice_Free	December
12/22/15	1.89E+06	6.28	Ice_Free	December
12/22/15	1.13E+06	6.05	Ice_Free	December
12/22/15	6.95E+05	5.84	Ice_Free	December
12/23/15	1.05E+07	7.02	Ice_Free	December
12/23/15	9.77E+06	6.99	Ice_Free	December
12/23/15	5.68E+06	6.75	Ice_Free	December
12/23/15	5.41E+06	6.73	Ice_Free	December
12/23/15	4.39E+06	6.64	Ice_Free	December
12/23/15	1.78E+06	6.25	Ice_Free	December
1/4/16	1.30E+07	7.11	Ice	January
1/4/16	8.52E+06	6.93	Ice	January
1/4/16	7.64E+06	6.88	Ice	January
1/4/16	4.55E+06	6.66	Ice	January
1/4/16	4.52E+06	6.66	Ice	January
1/4/16	3.15E+06	6.50	Ice	January
1/5/16	2.20E+09	9.34	Ice	January
1/5/16	1.51E+07	7.18	Ice	January
1/5/16	1.03E+07	7.01	Ice	January
1/5/16	7.91E+06	6.90	Ice	January
1/5/16	1.47E+05	5.17	Ice	January
1/5/16	3.47E+04	4.54	Ice	January
1/6/16	1.22E+07	7.09	Ice	January
1/6/16	9.57E+06	6.98	Ice	January
1/6/16	8.37E+06	6.92	Ice	January
1/6/16	5.35E+06	6.73	Ice	January
1/6/16	5.15E+06	6.71	Ice	January
1/6/16	1.20E+05	5.08	Ice	January
1/7/16	7.89E+08	8.90	Ice	January
1/7/16	1.32E+07	7.12	Ice	January
1/7/16	1.09E+07	7.04	Ice	January
1/8/16	3.70E+07	7.57	Ice	January
1/8/16	2.97E+07	7.47	Ice	January
1/8/16	2.00E+07	7.30	Ice	January
1/8/16	1.71E+07	7.23	Ice	January
1/8/16	1.38E+07	7.14	Ice	January
1/8/16	1.17E+07	7.07	Ice	January

1/11/16	2.97E+08	8.47	Ice	January
1/11/16	1.24E+07	7.09	Ice	January
1/11/16	1.12E+07	7.05	Ice	January
1/11/16	9.10E+06	6.96	Ice	January
1/11/16	5.75E+06	6.76	Ice	January
1/11/16	3.94E+06	6.60	Ice	January
1/12/16	1.35E+08	8.13	Ice	January
1/12/16	1.34E+07	7.13	Ice	January
1/12/16	1.15E+07	7.06	Ice	January
1/12/16	9.05E+03	3.96	Ice	January
1/12/16	7.87E+03	3.90	Ice	January
1/12/16	5.23E+03	3.72	Ice	January
1/13/16	1.31E+07	7.12	Ice	January
1/13/16	9.83E+06	6.99	Ice	January
1/13/16	7.93E+06	6.90	Ice	January
1/13/16	8.13E+03	3.91	Ice	January
1/13/16	4.64E+03	3.67	Ice	January
1/13/16	3.53E+03	3.55	Ice	January
1/14/16	3.86E+09	9.59	Ice	January
1/14/16	1.08E+07	7.03	Ice	January
1/14/16	9.61E+06	6.98	Ice	January
1/14/16	7.94E+06	6.90	Ice	January
1/14/16	4.27E+06	6.63	Ice	January
1/14/16	5.79E+03	3.76	Ice	January
1/15/16	1.26E+07	7.10	Ice	January
1/15/16	1.02E+07	7.01	Ice	January
1/15/16	8.80E+06	6.94	Ice	January
1/15/16	7.75E+06	6.89	Ice	January
1/15/16	6.31E+06	6.80	Ice	January
1/15/16	3.12E+06	6.49	Ice	January
1/18/16	2.44E+07	7.39	Ice	January
1/18/16	1.18E+07	7.07	Ice	January
1/18/16	1.08E+07	7.03	Ice	January
1/18/16	8.15E+06	6.91	Ice	January
1/18/16	2.93E+06	6.47	Ice	January
1/18/16	5.14E+04	4.71	Ice	January
1/19/16	6.92E+06	6.84	Ice	January
1/19/16	5.05E+06	6.70	Ice	January
1/19/16	5.03E+06	6.70	Ice	January

1/19/16	4.37E+06	6.64	Ice	January
1/19/16	2.16E+05	5.33	Ice	January
1/19/16	2.83E+04	4.45	Ice	January
1/20/16	1.31E+07	7.12	Ice	January
1/20/16	8.24E+06	6.92	Ice	January
1/20/16	7.82E+06	6.89	Ice	January
1/20/16	6.55E+06	6.82	Ice	January
1/20/16	6.54E+06	6.82	Ice	January
1/20/16	4.21E+06	6.62	Ice	January
1/21/16	1.47E+06	6.17	Ice	January
1/22/16	9.63E+07	7.98	Ice	January
1/22/16	9.25E+04	4.97	Ice	January
1/25/16	2.54E+06	6.40	Ice	January
1/25/16	1.04E+06	6.02	Ice	January
1/25/16	2.05E+05	5.31	Ice	January
1/25/16	1.64E+04	4.21	Ice	January
1/25/16	8.02E+02	2.90	Ice	January
1/26/16	6.28E+06	6.80	Ice	January
1/26/16	3.98E+06	6.60	Ice	January
1/26/16	2.39E+06	6.38	Ice	January
1/26/16	7.69E+05	5.89	Ice	January
1/26/16	5.10E+05	5.71	Ice	January
1/26/16	1.55E+04	4.19	Ice	January
1/27/16	3.19E+06	6.50	Ice	January
1/27/16	2.98E+06	6.47	Ice	January
1/27/16	2.23E+06	6.35	Ice	January
1/27/16	2.01E+06	6.30	Ice	January
1/27/16	9.95E+05	6.00	Ice	January
1/27/16	2.94E+04	4.47	Ice	January
1/28/16	3.45E+06	6.54	Ice	January
1/28/16	8.82E+05	5.95	Ice	January
1/28/16	5.65E+05	5.75	Ice	January
1/28/16	3.14E+05	5.50	Ice	January
1/28/16	2.27E+05	5.36	Ice	January
1/28/16	1.14E+04	4.06	Ice	January
1/29/16	4.08E+06	6.61	Ice	January
1/29/16	2.34E+06	6.37	Ice	January
1/29/16	1.43E+06	6.16	Ice	January
1/29/16	9.62E+05	5.98	Ice	January

1/29/16	9.31E+05	5.97	Ice	January
1/29/16	5.67E+05	5.75	Ice	January
2/1/16	3.12E+06	6.49	Ice	February
2/1/16	2.63E+06	6.42	Ice	February
2/1/16	1.35E+06	6.13	Ice	February
2/1/16	7.36E+05	5.87	Ice	February
2/1/16	7.07E+05	5.85	Ice	February
2/1/16	2.09E+05	5.32	Ice	February
2/2/16	1.14E+06	6.06	Ice	February
2/2/16	6.49E+05	5.81	Ice	February
2/2/16	3.03E+05	5.48	Ice	February
2/2/16	2.60E+05	5.41	Ice	February
2/2/16	2.44E+05	5.39	Ice	February
2/2/16	1.67E+05	5.22	Ice	February
2/3/16	1.62E+06	6.21	Ice	February
2/3/16	1.54E+06	6.19	Ice	February
2/3/16	1.27E+06	6.10	Ice	February
2/3/16	1.18E+06	6.07	Ice	February
2/3/16	3.05E+05	5.48	Ice	February
2/3/16	9.70E+04	4.99	Ice	February
2/4/16	1.36E+06	6.13	Ice	February
2/4/16	8.04E+05	5.91	Ice	February
2/4/16	6.96E+05	5.84	Ice	February
2/4/16	4.99E+05	5.70	Ice	February
2/4/16	4.50E+05	5.65	Ice	February
2/4/16	1.32E+05	5.12	Ice	February
2/5/16	2.50E+06	6.40	Ice	February
2/5/16	5.68E+05	5.75	Ice	February
2/5/16	4.66E+04	4.67	Ice	February
2/5/16	1.29E+04	4.11	Ice	February
2/5/16	1.19E+04	4.08	Ice	February
2/5/16	7.19E+03	3.86	Ice	February
2/8/16	3.29E+06	6.52	Ice	February
2/8/16	2.59E+06	6.41	Ice	February
2/8/16	1.35E+06	6.13	Ice	February
2/8/16	8.05E+05	5.91	Ice	February
2/8/16	6.16E+03	3.79	Ice	February
2/8/16	1.57E+03	3.20	Ice	February
2/9/16	1.28E+06	6.11	Ice	February

2/9/16	7.90E+05	5.90	Ice	February
2/9/16	4.45E+05	5.65	Ice	February
2/9/16	2.23E+03	3.35	Ice	February
2/11/16	3.26E+06	6.51	Ice	February
2/11/16	2.59E+06	6.41	Ice	February
2/11/16	2.36E+06	6.37	Ice	February
2/11/16	2.16E+06	6.33	Ice	February
2/11/16	1.87E+06	6.27	Ice	February
2/11/16	1.53E+06	6.18	Ice	February
2/12/16	4.05E+05	5.61	Ice	February
2/12/16	3.74E+05	5.57	Ice	February
2/12/16	2.28E+05	5.36	Ice	February
2/12/16	4.94E+04	4.69	Ice	February
2/12/16	3.87E+02	2.59	Ice	February
2/15/16	3.86E+06	6.59	Ice	February
2/15/16	3.49E+06	6.54	Ice	February
2/15/16	3.00E+06	6.48	Ice	February
2/15/16	2.48E+06	6.39	Ice	February
2/15/16	9.30E+05	5.97	Ice	February
2/15/16	8.43E+05	5.93	Ice	February
2/16/16	3.09E+06	6.49	Ice	February
2/16/16	2.16E+06	6.33	Ice	February
2/16/16	1.88E+06	6.27	Ice	February
2/16/16	1.78E+06	6.25	Ice	February
2/16/16	1.49E+06	6.17	Ice	February
2/16/16	9.38E+05	5.97	Ice	February
2/18/16	2.55E+06	6.41	Ice	February
2/18/16	2.25E+06	6.35	Ice	February
2/18/16	2.09E+06	6.32	Ice	February
2/18/16	1.65E+06	6.22	Ice	February
2/18/16	1.43E+06	6.16	Ice	February
2/18/16	8.22E+05	5.91	Ice	February
2/19/16	5.45E+06	6.74	Ice	February
2/19/16	4.75E+06	6.68	Ice	February
2/19/16	3.06E+06	6.49	Ice	February
2/19/16	3.05E+06	6.48	Ice	February
2/19/16	2.87E+06	6.46	Ice	February
2/19/16	1.15E+06	6.06	Ice	February
2/22/16	4.47E+06	6.65	Ice	February

2/22/16	3.65E+06	6.56	Ice	February
2/22/16	1.10E+06	6.04	Ice	February
2/22/16	7.18E+05	5.86	Ice	February
2/22/16	6.01E+04	4.78	Ice	February
2/22/16	3.16E+04	4.50	Ice	February
2/23/16	8.64E+06	6.94	Ice	February
2/23/16	4.58E+06	6.66	Ice	February
2/23/16	3.98E+06	6.60	Ice	February
2/23/16	3.49E+06	6.54	Ice	February
2/23/16	2.35E+06	6.37	Ice	February
2/23/16	1.95E+06	6.29	Ice	February
2/25/16	2.01E+06	6.30	Ice	February
2/25/16	1.71E+06	6.23	Ice	February
2/25/16	1.28E+06	6.11	Ice	February
2/25/16	1.01E+06	6.00	Ice	February
2/25/16	4.70E+04	4.67	Ice	February
2/25/16	3.96E+04	4.60	Ice	February
2/26/16	3.95E+06	6.60	Ice	February
2/26/16	2.11E+06	6.32	Ice	February
2/26/16	1.75E+06	6.24	Ice	February
2/26/16	1.44E+06	6.16	Ice	February
2/26/16	1.35E+06	6.13	Ice	February
2/26/16	9.80E+05	5.99	Ice	February
2/29/16	3.68E+06	6.57	Ice	February
2/29/16	2.88E+06	6.46	Ice	February
2/29/16	2.37E+06	6.37	Ice	February
2/29/16	2.00E+06	6.30	Ice	February
2/29/16	9.77E+05	5.99	Ice	February
2/29/16	7.54E+05	5.88	Ice	February
3/2/16	3.06E+06	6.49	Ice	March
3/2/16	1.42E+06	6.15	Ice	March
3/2/16	1.33E+06	6.12	Ice	March
3/2/16	1.12E+06	6.05	Ice	March
3/2/16	8.36E+05	5.92	Ice	March
3/2/16	2.98E+05	5.47	Ice	March
3/3/16	4.05E+06	6.61	Ice	March
3/3/16	2.02E+06	6.31	Ice	March
3/3/16	1.80E+06	6.26	Ice	March
3/3/16	1.48E+06	6.17	Ice	March

3/3/16	1.02E+06	6.01	Ice	March
3/3/16	9.72E+05	5.99	Ice	March
3/4/16	3.99E+06	6.60	Ice	March
3/4/16	3.47E+06	6.54	Ice	March
3/4/16	3.44E+06	6.54	Ice	March
3/4/16	3.12E+06	6.49	Ice	March
3/4/16	2.96E+04	4.47	Ice	March
3/4/16	1.24E+04	4.09	Ice	March
3/7/16	4.57E+06	6.66	Ice	March
3/7/16	4.27E+06	6.63	Ice	March
3/7/16	2.67E+06	6.43	Ice	March
3/7/16	2.59E+06	6.41	Ice	March
3/7/16	2.53E+06	6.40	Ice	March
3/7/16	2.42E+06	6.38	Ice	March
3/8/16	4.87E+06	6.69	Ice	March
3/8/16	4.04E+06	6.61	Ice	March
3/8/16	3.91E+06	6.59	Ice	March
3/8/16	2.35E+06	6.37	Ice	March
3/8/16	1.95E+04	4.29	Ice	March
3/8/16	1.73E+04	4.24	Ice	March
3/9/16	5.84E+06	6.77	Ice	March
3/9/16	3.95E+06	6.60	Ice	March
3/9/16	1.71E+06	6.23	Ice	March
3/9/16	1.28E+06	6.11	Ice	March
3/9/16	6.15E+05	5.79	Ice	March
3/9/16	2.75E+05	5.44	Ice	March
3/10/16	4.03E+06	6.61	Ice_Free	March
3/10/16	3.74E+06	6.57	Ice_Free	March
3/10/16	3.58E+06	6.55	Ice_Free	March
3/10/16	2.87E+06	6.46	Ice_Free	March
3/10/16	2.43E+06	6.39	Ice_Free	March
3/10/16	2.05E+06	6.31	Ice_Free	March
3/11/16	5.12E+06	6.71	Ice_Free	March
3/11/16	3.82E+06	6.58	Ice_Free	March
3/11/16	3.79E+06	6.58	Ice_Free	March
3/11/16	3.38E+06	6.53	Ice_Free	March
3/11/16	2.94E+06	6.47	Ice_Free	March
3/11/16	2.07E+06	6.32	Ice_Free	March
3/14/16	6.61E+06	6.82	Ice_Free	March

3/14/16	5.66E+06	6.75	Ice_Free	March
3/14/16	4.50E+06	6.65	Ice_Free	March
3/14/16	3.31E+06	6.52	Ice_Free	March
3/14/16	2.09E+06	6.32	Ice_Free	March
3/14/16	1.86E+06	6.27	Ice_Free	March
3/15/16	5.01E+06	6.70	Ice_Free	March
3/15/16	4.39E+06	6.64	Ice_Free	March
3/15/16	2.90E+06	6.46	Ice_Free	March
3/15/16	2.77E+06	6.44	Ice_Free	March
3/15/16	2.76E+06	6.44	Ice_Free	March
3/15/16	2.64E+06	6.42	Ice_Free	March
3/21/16	1.27E+07	7.10	Ice_Free	March
3/21/16	1.21E+07	7.08	Ice_Free	March
3/21/16	6.49E+06	6.81	Ice_Free	March
3/21/16	5.18E+06	6.71	Ice_Free	March
3/21/16	4.73E+06	6.67	Ice_Free	March
3/21/16	4.12E+06	6.61	Ice_Free	March
3/22/16	1.09E+07	7.04	Ice_Free	March
3/22/16	1.07E+07	7.03	Ice_Free	March
3/22/16	7.66E+06	6.88	Ice_Free	March
3/22/16	7.02E+06	6.85	Ice_Free	March
3/22/16	4.44E+06	6.65	Ice_Free	March
3/22/16	3.68E+06	6.57	Ice_Free	March
3/23/16	9.02E+06	6.96	Ice_Free	March
3/23/16	7.20E+06	6.86	Ice_Free	March
3/23/16	6.79E+06	6.83	Ice_Free	March
3/23/16	6.64E+06	6.82	Ice_Free	March
3/23/16	4.89E+06	6.69	Ice_Free	March
3/23/16	3.09E+06	6.49	Ice_Free	March
3/24/16	2.23E+07	7.35	Ice_Free	March
3/24/16	1.96E+07	7.29	Ice_Free	March
3/24/16	1.71E+07	7.23	Ice_Free	March
3/24/16	9.09E+06	6.96	Ice_Free	March
3/24/16	1.71E+04	4.23	Ice_Free	March
3/24/16	1.41E+04	4.15	Ice_Free	March
3/28/16	3.77E+07	7.58	Ice_Free	March
3/28/16	3.03E+07	7.48	Ice_Free	March
3/28/16	2.98E+07	7.47	Ice_Free	March
3/28/16	2.54E+07	7.40	Ice_Free	March

3/28/16	1.51E+07	7.18	Ice_Free	March
3/28/16	3.77E+06	6.58	Ice_Free	March
3/29/16	2.42E+07	7.38	Ice_Free	March
3/29/16	1.42E+07	7.15	Ice_Free	March
3/29/16	1.18E+07	7.07	Ice_Free	March
3/29/16	8.76E+06	6.94	Ice_Free	March
3/29/16	7.06E+06	6.85	Ice_Free	March
3/29/16	5.55E+06	6.74	Ice_Free	March
3/31/16	3.91E+07	7.59	Ice_Free	March
3/31/16	1.66E+07	7.22	Ice_Free	March
3/31/16	1.24E+07	7.09	Ice_Free	March
3/31/16	1.01E+07	7.00	Ice_Free	March
3/31/16	3.24E+06	6.51	Ice_Free	March
3/31/16	3.24E+06	6.51	Ice_Free	March
4/5/16	1.32E+07	7.12	Ice_Free	April
4/5/16	1.05E+07	7.02	Ice_Free	April
4/5/16	8.43E+06	6.93	Ice_Free	April
4/5/16	5.02E+06	6.70	Ice_Free	April
4/5/16	4.81E+06	6.68	Ice_Free	April
4/5/16	1.99E+05	5.30	Ice_Free	April
4/7/16	2.03E+07	7.31	Ice_Free	April
4/7/16	1.98E+07	7.30	Ice_Free	April
4/7/16	1.67E+07	7.22	Ice_Free	April
4/7/16	1.55E+07	7.19	Ice_Free	April
4/7/16	1.45E+07	7.16	Ice_Free	April
4/7/16	1.02E+07	7.01	Ice_Free	April
4/8/16	3.10E+07	7.49	Ice_Free	April
4/8/16	1.62E+07	7.21	Ice_Free	April
4/8/16	1.38E+07	7.14	Ice_Free	April
4/8/16	1.37E+07	7.14	Ice_Free	April
4/8/16	8.58E+06	6.93	Ice_Free	April
4/8/16	5.35E+06	6.73	Ice_Free	April
4/12/16	2.96E+07	7.47	Ice_Free	April
4/12/16	2.23E+07	7.35	Ice_Free	April
4/12/16	6.10E+06	6.79	Ice_Free	April
4/12/16	5.64E+06	6.75	Ice_Free	April
4/12/16	5.10E+06	6.71	Ice_Free	April
4/12/16	4.38E+06	6.64	Ice_Free	April
4/13/16	8.58E+06	6.93	Ice_Free	April

4/13/16	8.22E+06	6.91	Ice_Free	April
4/13/16	6.37E+06	6.80	Ice_Free	April
4/13/16	4.73E+06	6.67	Ice_Free	April
4/13/16	4.55E+06	6.66	Ice_Free	April
4/14/16	1.62E+07	7.21	Ice_Free	April
4/14/16	1.60E+07	7.20	Ice_Free	April
4/14/16	1.31E+07	7.12	Ice_Free	April
4/14/16	9.28E+06	6.97	Ice_Free	April
4/14/16	8.38E+06	6.92	Ice_Free	April
4/14/16	2.21E+06	6.34	Ice_Free	April
4/18/16	7.21E+06	6.86	Ice_Free	April
4/18/16	5.62E+06	6.75	Ice_Free	April
4/18/16	4.98E+06	6.70	Ice_Free	April
4/18/16	4.65E+06	6.67	Ice_Free	April
4/18/16	2.56E+06	6.41	Ice_Free	April
4/18/16	1.37E+06	6.14	Ice_Free	April
4/19/16	6.90E+06	6.84	Ice_Free	April
4/19/16	6.68E+06	6.82	Ice_Free	April
4/19/16	4.93E+06	6.69	Ice_Free	April
4/19/16	3.98E+06	6.60	Ice_Free	April
4/19/16	3.74E+06	6.57	Ice_Free	April
4/19/16	3.22E+06	6.51	Ice_Free	April
4/20/16	1.64E+07	7.21	Ice_Free	April
4/20/16	1.27E+07	7.10	Ice_Free	April
4/20/16	1.25E+07	7.10	Ice_Free	April
4/20/16	1.04E+07	7.02	Ice_Free	April
4/20/16	7.80E+06	6.89	Ice_Free	April
4/20/16	2.66E+05	5.42	Ice_Free	April
